# Adamantyl Ethanone Pyridyl Derivatives: Potent and Selective Inhibitors of Human 11β-Hydroxysteroid Dehydrogenase Type 1

**DOI:** 10.1002/cmdc.201100182

**Published:** 2011-06-28

**Authors:** Xiangdong Su, Fabienne Pradaux-Caggiano, Nigel Vicker, Mark P Thomas, Heather Halem, Michael D Culler, Barry V L Potter

**Affiliations:** [a] Medicinal Chemistry, Department of Pharmacy and Pharmacology, University of BathBath, BA2 7AY (UK), Fax: (+44) 1225 386114; [b] IPSEN, Biomeasure Inc.27 Maple Street, Milford, MA 01757 (USA)

**Keywords:** 11β-HSD1, adamantyl ethanones, diabetics, hydroxysteroid dehydrogenases, inhibitors, metabolic syndrome

## Abstract

Elevated levels of active glucocorticoids have been implicated in the development of several phenotypes of metabolic syndrome, such as type 2 diabetes and obesity. 11β-Hydroxysteroid dehydrogenase type 1 (11β-HSD1) catalyses the intracellular conversion of inactive cortisone to cortisol. Selective 11β-HSD1 inhibitors have shown beneficial effects in various conditions, including diabetes, dyslipidemia and obesity. A series of adamantyl ethanone pyridyl derivatives has been identified, providing potent and selective inhibitors of human 11β-HSD1. Lead compounds display low nanomolar inhibition against human and mouse 11β-HSD1 and are selective for this isoform, with no activity against 11β-HSD2 and 17β-HSD1. Structure–activity relationship studies reveal that an unsubstituted pyridine tethered to an adamantyl ethanone motif through an ether or sulfoxide linker provides a suitable pharmacophore for activity. The most potent inhibitors have IC_50_ values around 34–48 nm against human 11β-HSD1, display reasonable metabolic stability in human liver microsomes, and weak inhibition of key human CYP450 enzymes.

## Introduction

Metabolic syndrome refers to a cluster of metabolic disorders, including insulin resistance, hyperglycemia, visceral obesity, hypertension and dyslipidemia. Combinations of these conditions represent major risk factors for developing cardiovascular disease and type 2 diabetes.[1, 2] The rapid increase in the prevalence of cardiovascular disease and type 2 diabetes demands novel and effective approaches for the prevention and treatment of the syndrome. Previous studies have implicated excessive glucocorticoid action in the development of several phenotypes associated with metabolic syndrome.[3–5] Abnormal glucocorticoid receptor (GR) signalling is associated with insulin and leptin resistance, leading to the development of type 2 diabetes, obesity and cardiovascular disorders.[6, 7] GR activation stimulates hepatic glucose production, antagonises insulin secretion from pancreatic β-cells and insulin-mediated glucose uptake in peripheral tissues,[8–11] and it also promotes lipolysis and fatty acid mobilisation.[12]

Metabolic syndrome shares similar characteristics with symptoms of Cushing’s syndrome, a systemic glucocorticoid excess condition.[13] As the metabolic abnormalities in Cushing’s syndrome can be improved to a certain degree by reducing the excessive glucocorticoid action,[14–16] the similarities between phenotypes of metabolic syndrome and Cushing’s syndrome suggest the possibility of treating the individual indications of metabolic syndrome by glucocorticoid activity suppression.

Systemic glucocorticoid levels are generally normal in patients with common forms of obesity or overweight type 2 diabetics.[17] Since GR signalling depends not only on the circulating glucocorticoid level, but also on the prereceptor activation of glucocorticoid within cells, it is speculated that the intracellular glucocorticoid concentration is responsible for metabolic disorders. The intracellular metabolism of glucocorticoid is mediated by 11β-hydroxysteroid dehydrogenase isozymes (11β-HSDs), which are microsomal enzymes from the short-chain dehydrogenase/reductase family. The 11β-hydroxysteroid dehydrogenase type 1 (11β-HSD1), highly expressed in liver, adipose tissue and the central nervous system, acts as an NADPH-dependent reductase, converting cortisone in humans to the active glucocorticoid cortisol. The prereceptor activation of cortisone mediated by 11β-HSD1 provides a mechanism for specific tissues to produce intracellular, nonadrenal cortisol, thereby locally amplifying the glucocorticoid action.[18] Conversely, the 11β-HSD2 isoform is exclusively NAD^+^ dependent and mainly expressed in mineralocorticoid target tissues, such as the cortical collection ducts of the kidney and the distal colon. 11β-HSD2 catalyses the transformation of cortisol to inactive cortisone and reduces the local concentration of cortisol in specific tissues. This mechanism prevents activation of the by cortisol in renal cortical collecting ducts and distal colon. Reduced 11β-HSD2 function may result in sodium retention, hypokalemia and hypertension.[19–21]

The correlation between 11β-HSD1 activity, obesity and diabetes has also been validated with genetically modified rodent models. Transgenic mice with fat-tissue-specific overexpression of 11β-HSD1 develop symptoms of insulin-resistant diabetes, hyperlipidemia and visceral obesity.[22, 23] In contrast, studies with 11β-HSD1 knock-out mice demonstrate that these animals resist stress-induced hyperglycaemia, diet-induced obesity, and have decreased cholesterol and triglyceride levels.[24, 25] Similarly, specific overexpression of 11β-HSD2 in adipose tissue in transgenic mice also results in increased insulin sensitivity, glucose tolerance, and resistance to body weight gain on a high-fat diet.[26] 11β-HSD1 activity suppression in animal models with selective inhibitors was found to provide beneficial effects in various indications of metabolic syndrome.[27–29] Moreover, clinical studies suggest that inhibition of 11β-HSD1 with carbenoxolone, a nonselective inhibitor, increases hepatic insulin sensitivity and decreases glucose production.[30, 31]

**Figure 1:**
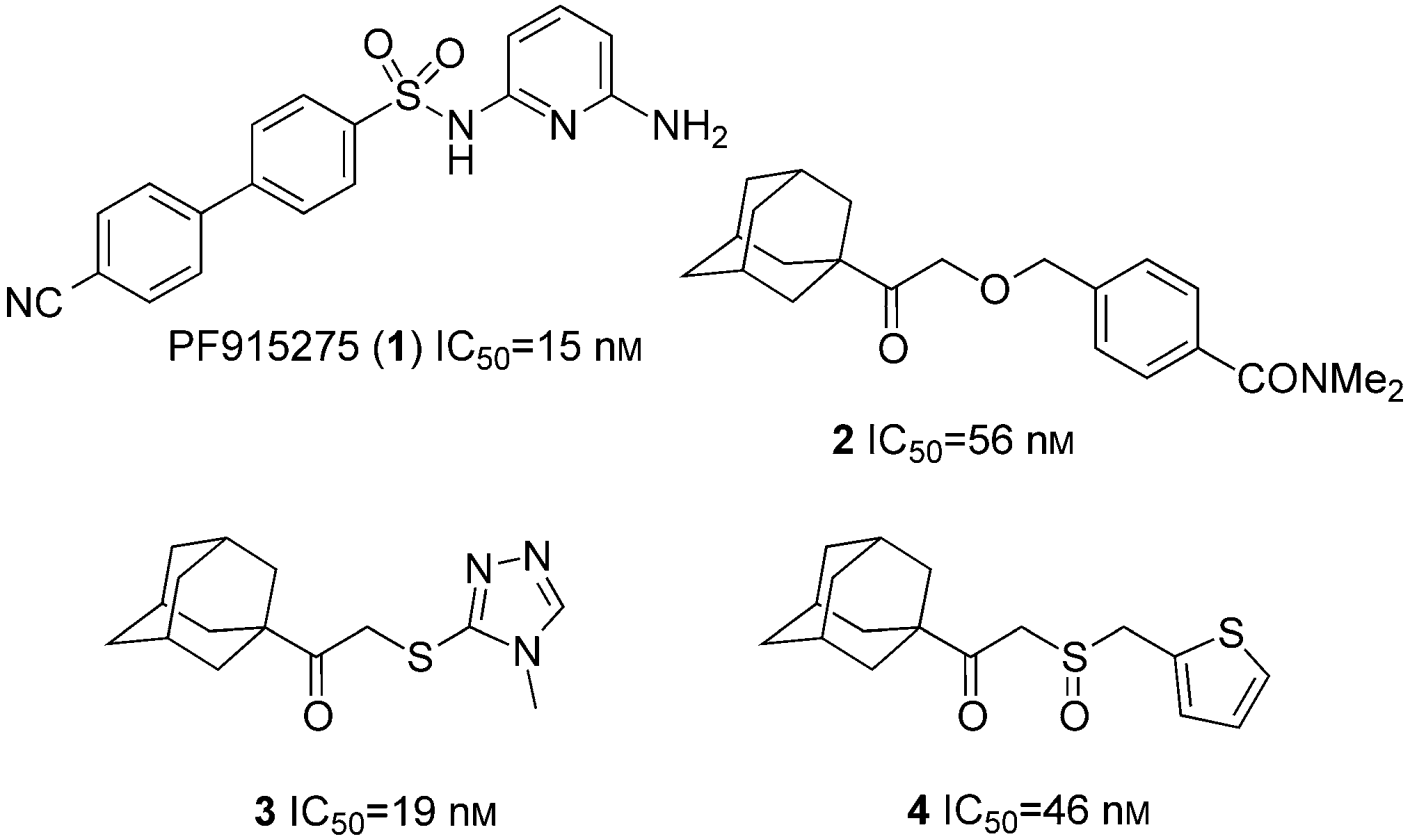
Known potent inhibitors of 11β-HSD1. The inhibitory activities of compounds 1–4 were measured in HEK293 cells transfected with the human *HSD11B1* gene.[38, 41–42]

The concept of treating type 2 diabetes, obesity, and other metabolic abnormalities through selective inhibition of 11β-HSD1 activity with small-molecule inhibitors has attracted considerable interest in the pharmaceutical industry over the last decade.[32–36] Newly discovered potent and selective 11β-HSD1 inhibitors have been extensively reviewed.[32–37] PF915275 (**1**; Figure 1), an inhibitor developed by Pfizer, has shown modest 11β-HSD1 inhibition in a clinical study.[38] Results from a positive proof-of-concept clinical study of 11β-HSD1 inhibitor INCB013739, developed by Incyte, provide substantial evidence that inhibition of 11β-HSD1 can be a viable treatment of type 2 diabetes. INCB013739 treatment of type 2 diabetes mellitus patients who failed on metformin monotherapy show significantly improved hepatic and peripheral insulin sensitivity and reduced haemoglobin A1c and fasting plasma glucose levels. In patients with hyperlipidaemia or hypertriglyceridaemia, treatment with INCB013739 also lowers triglyceride and cholesterol levels.[39, 40]

We previously identified some adamantyl ethanone derivatives as potent inhibitors of human 11β-HSD1.[41, 42] Compounds **2**–**4** exhibit high inhibitory activity with nanomolar IC_50_ values when examined in a HEK293 cell line stably transfected with the *HSD11B1* gene (Figure 1). These compounds also show reasonable metabolic stability when incubated with human liver microsomes. To improve potency, pharmacokinetic properties and physicochemical properties, we performed further optimisation on this series of compound using structure-based design.[43] We synthesised compounds containing a pyridyl ring tethered to an adamantyl ethanone motif through an oxygen, sulfur, sulfoxide, sulfone or amide linker, and examined their inhibitory activity against human 11β-HSD1. Selected potent compounds were also tested for activity against mouse 11β-HSD1. Their selectivity for 11β-HSD1 over 11β-HSD2 and 17β-HSD1 was also examined.

## Results and Discussion

### Chemistry

**Scheme 1:**
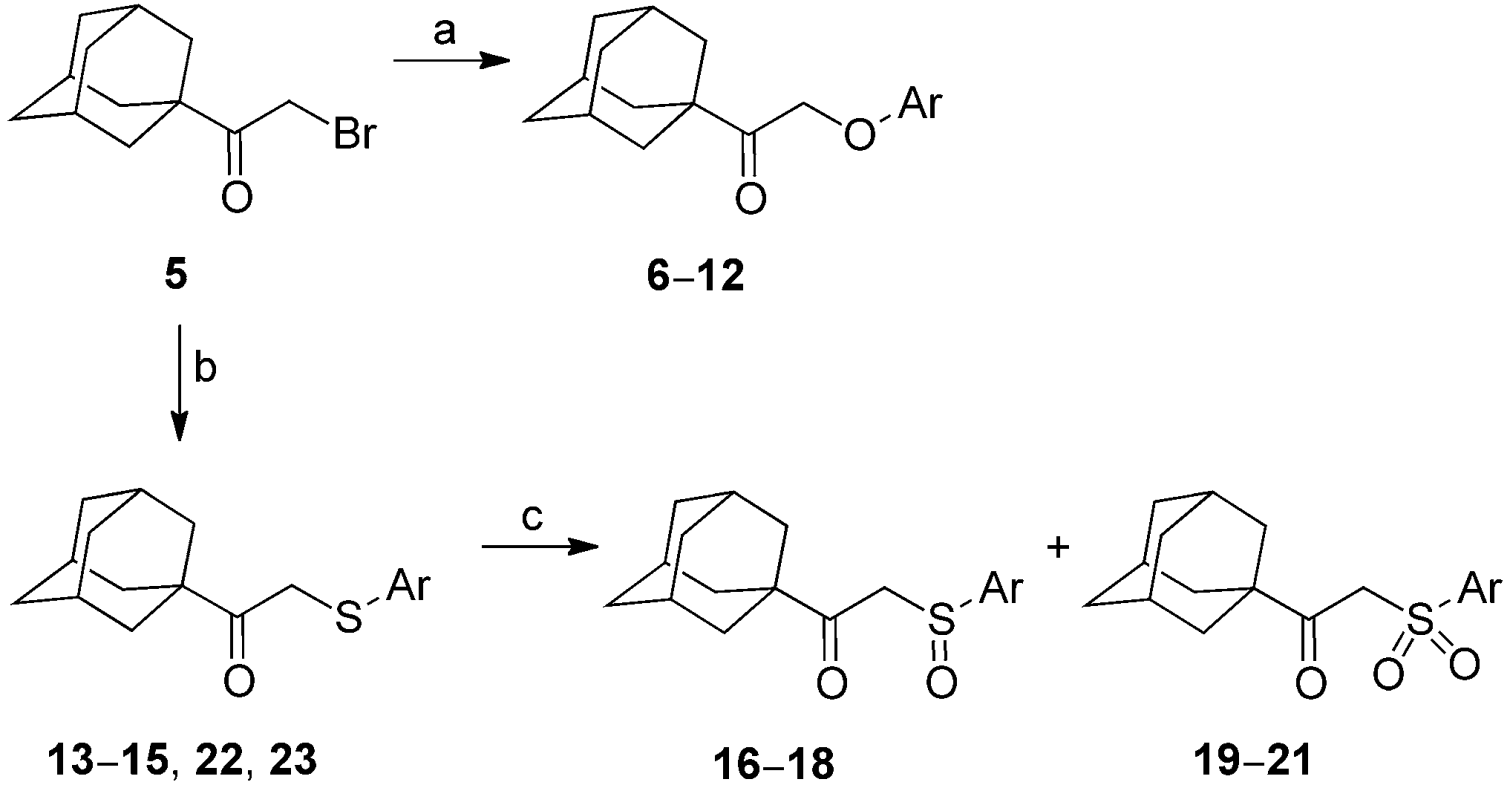
Synthesis of the adamantyl ethanone compounds (for Ar, see Tables 1–3). *Reagents and conditions*: a) ArOH, K_2_CO_3_, acetone, RT; b) ArSH, Et_3_N, CH_3_CN, RT; c) *m*-CPBA, CH_2_Cl_2_, −10→0 °C.

Adamantyl ethanones **6**–**12**, with an oxygen linker, were prepared by a nucleophilic coupling reaction between the corresponding pyridinol and 1-adamantyl bromomethyl ketone (**5**) under basic conditions (Scheme 1). Compound **5** was reacted with commercially available pyridine thiol using triethylamine in acetonitrile to give the corresponding sulfur linker compounds (**13**–**15**, **22** and **23**). Further oxidation of these compounds with *meta*-chloroperbenzoic acid (*m-*CPBA) at low temperature generates in one step both sulfoxide (**16**–**18**) and sulfone (**19**–**21**), which can be separated using flash chromatography (Scheme 1).

**Scheme 2:**
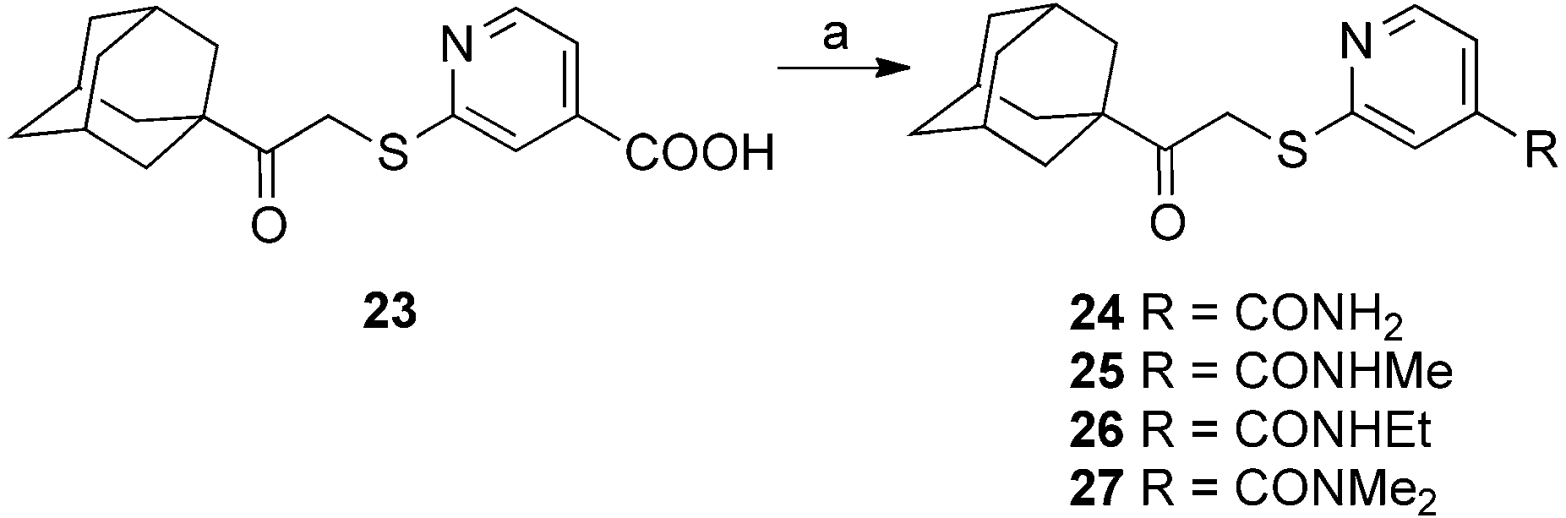
Synthesis of pyridyl carboxamide derivatives **24**–**27**. *Reagents and conditions*: a) EDCI, DMAP, Et_3_N, CH_2_Cl_2_, corresponding amine, RT, overnight.

Starting from **23**, the pyridyl carboxamide derivatives **24**–**27** were synthesised with amide formation reactions using 1-ethyl-3-(3-dimethylaminopropyl) carbodiimide (EDCI) and 4-dimethylaminopyridine (DMAP) as catalysts (Scheme 2).

**Scheme 3:**
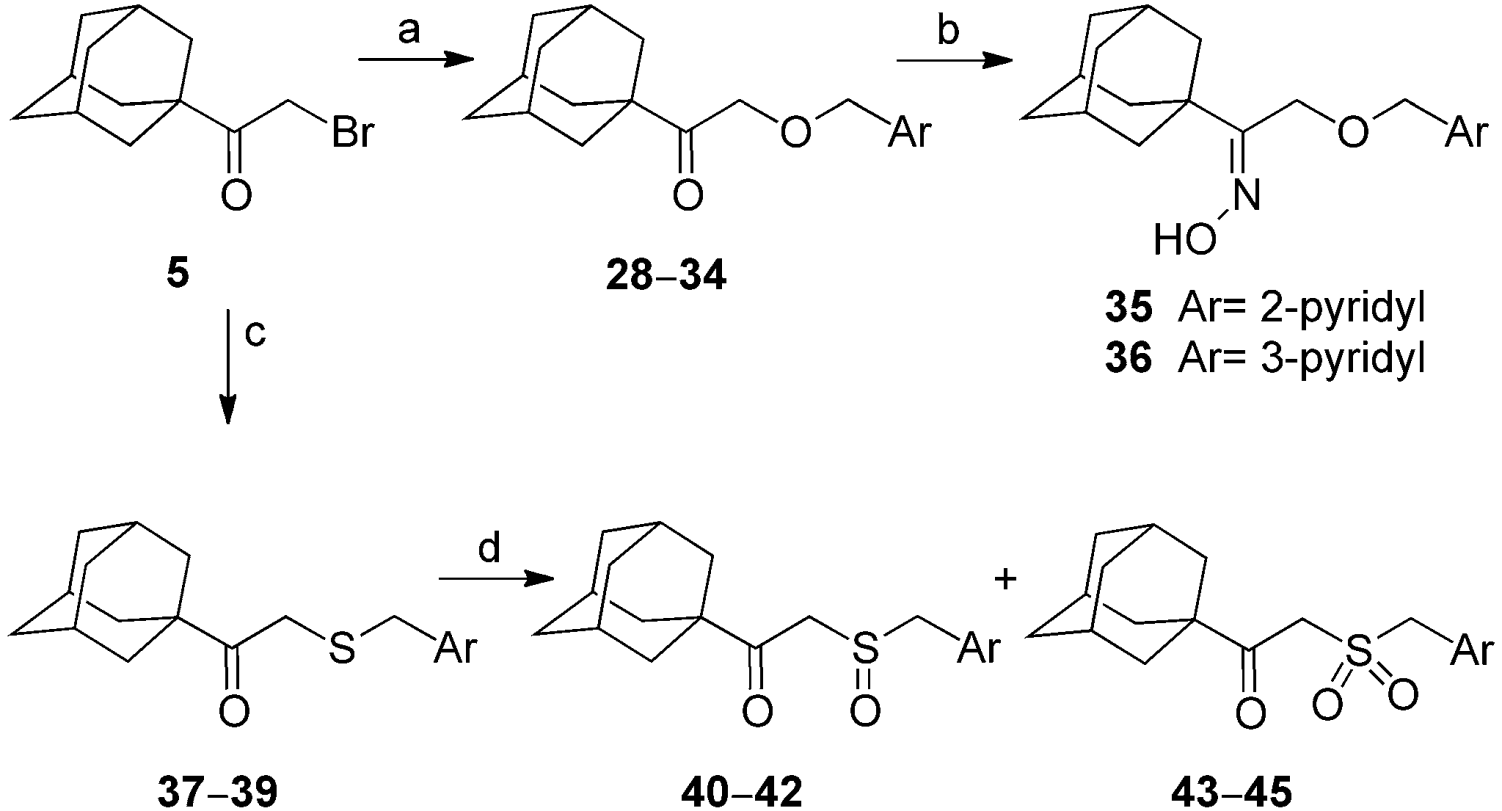
Synthesis of compounds with an extended linker (for Ar, see Tables 4 and 5). *Reagents and conditions*: a) ArCH_2_OH, NaH, THF, 0 °C; b) NH_2_OH⋅HCl, Et_3_N, EtOH, reflux, overnight; c) 1. ArCH_2_SC(NH)NH_2_⋅2HCl, NaOH, reflux; 2. Et_3_N, CH_3_CN, RT; d) *m*-CPBA, CH_2_Cl_2_, −10→0 °C.

The extended ether linker compounds **28**–**34** were synthesised from compound **5** and the corresponding pyridyl methyl alcohol under basic conditions using sodium hydride in a tetrahydrofuran (THF) solution (Scheme 3). Compounds **28** and **31** were transformed into their oxime derivatives **35** and **36**, respectively, as illustrated in Scheme 3. The target compounds with an extended sulfur linker were prepared by two different routes. Chloromethylpyridine was reacted with thiourea in ethanol to make the corresponding pyridinylmethyl carbamimidothioate dihydrochloride; the intermediate was then hydrolysed under basic conditions to generate the corresponding thiol, which was then transformed in situ into compounds **37**–**39** through a nucleophilic substitution reaction with compound **5** (Scheme 3).

**Scheme 4:**
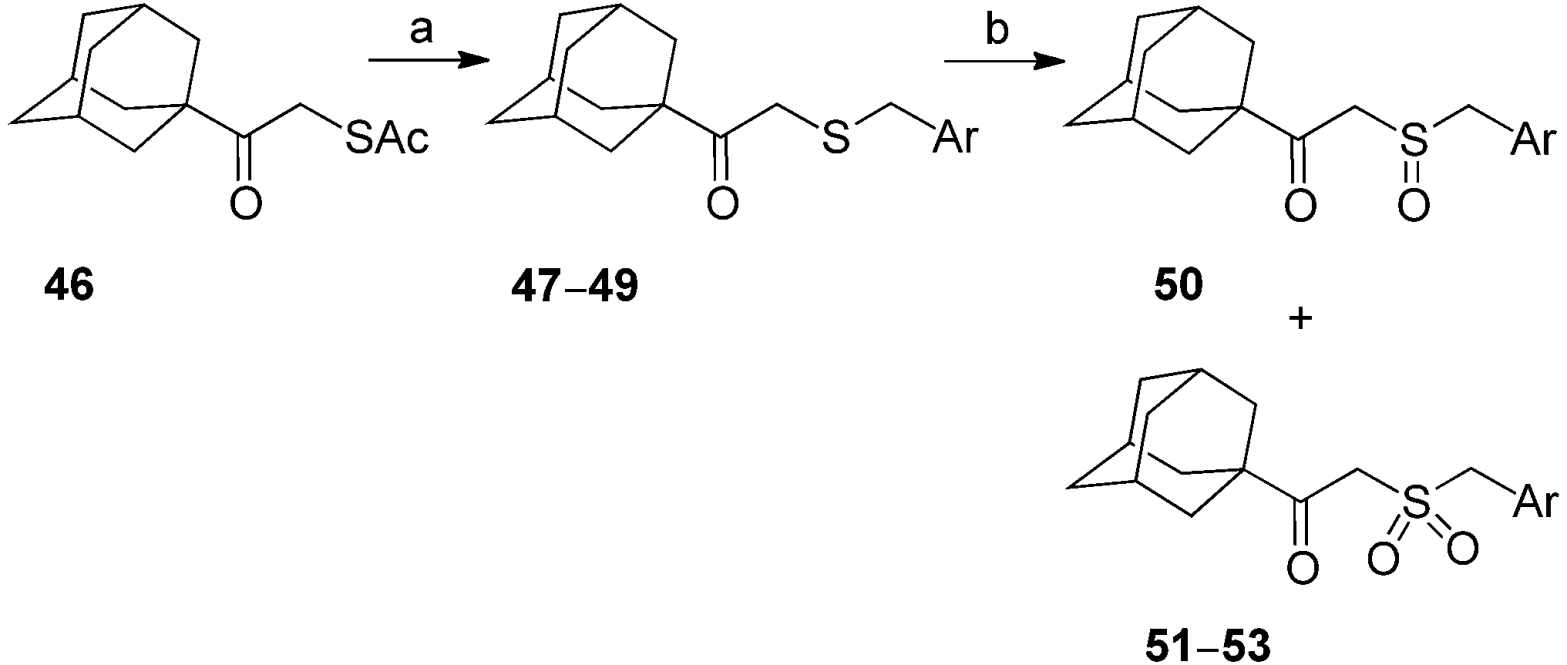
Synthesis of substituted pyridyl derivatives **47**–**53** (for Ar, see Table 6). *Reagents and conditions*: a) 1. 1 n NaOH, acetone, RT; 2. ArCH_2_Cl, Et_3_N, CH_3_CN, RT; b) *m*-CPBA, CH_2_Cl_2_, −10→0 °C.

Compound **46**, obtained by reacting **5** with potassium ethanethioate, was hydrolysed in basic conditions to produce the intermediate 2-mercapto-1-adamantylethanone, which was reacted in situ with the corresponding chloromethylpyridine to afford compounds **47**–**49** (Scheme 4). Sulfoxides **40**–**42**, **50** and sulfones **43**–**45**, **51**–**53** were obtained by *m*-CPBA oxidation of their corresponding thioether compound.

**Scheme 5:**
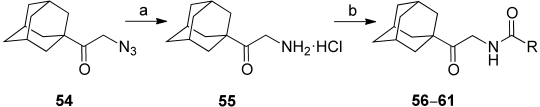
Synthesis of amide linker compounds **56**–**61** (for R, see Table 7). *Reagents and conditions*: a) H_2_, 5% Pd/C, CH_3_OH, 36 % HCl, RT, 6 h, 95 %; b) RCOOH, EDCI, DMAP, Et_3_N, CH_2_Cl_2_, RT, overnight.

The target compounds with an amide linker were prepared from the azido ethanone **54**, which was obtained through a nucleophilic substitution of compound **5** with sodium azide. Hydrogenation of **54** under acidic conditions gave amine intermediate **55**, which was reacted with the corresponding carboxylic acid to generate the target compounds **56**–**61** (Scheme 5).

### Structure–activity relationships

We previously identified potent and selective 11β-HSD1 inhibitors from compounds with an adamantyl ethanone core structure. Lead compounds **2**–**4**, with a substituted phenyl ring or 5-membered aromatic heterocycle tethered to the adamantyl ethanone moiety through an oxygen or sulfur linker, display selective inhibitory activity against both human and mouse 11β-HSD1.[41] To further optimise this series of 11β-HSD1 inhibitors, we designed and synthesised novel compounds based on the same core structure but with a pyridyl ring attached to the ethanone motif. The target compounds were tested for their inhibition against 11β-HSD1 in a HEK293 cell line transfected with the *HSD11B1* gene. As inhibitory activity was tested in intact cells, the result represents the cumulative effects of a compound’s cellular transportation, metabolism and binding affinity to 11β-HSD1.

In the ethanone ether series, compound **6**, with a 6-methyl-2-pyridyl ring, exhibited only moderate activity (IC_50_=3.1 μm). Replacing the 6-methyl group with an electron-withdrawing chloro or trifluoro group at either the 6- or 5-position resulted in loss of activity (compounds **7**, **8**). However, the 6-methyl-3-pyridyl compound **11** displays greatly enhanced activity with an IC_50_ value of 81 nm, a 38-fold improvement compared with the 2-pyridyl analogue **6**, suggesting that the nitrogen position is critical with such a linker system. More interestingly, the nonsubstituted compound **12** shows even greater inhibition (IC_50_=27 nm), indicating that the methyl group hinders the binding of the pyridyl nitrogen in the active site. This observation is in agreement to what was found in the 5-membered heterocyclic series, suggesting limited steric and/or electronic requirements around the aromatic ring (Table 1).

**Table 1:** Cellular 11β-HSD1 inhibition by compounds **6**–**12**

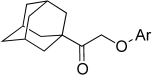		

Compd	Ar[a]	IC_50_[b] [nm]
**6**	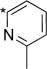	3109
**7**	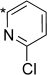	28 %^[c]^
**8**	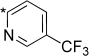	40 %^[c]^
**9**	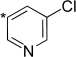	30 %^[c]^
**10**	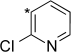	1362
**11**	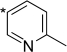	81
**12**	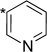	27

[a] * Indicates point of attachment.

[b] IC_50_ values are reported as the mean value of three measurements with variance less than 20 %.

[c] Percent inhibition measured at 1 μm and reported as the mean value of two measurements.

Replacing the oxygen linker with a sulfur atom in the same core structure generates some highly potent inhibitors. Both 2-pyridyl (**13**) and 4-pyridyl (**15**) derivatives display high potency with IC_50_ values of ∼60 nm (Table 2). However, the 6-methyl substituent has a negative effect on the activity, as compound **14** shows only 70 % inhibition at 1 μm. Interestingly, the corresponding sulfoxide linker derivatives **16**–**18** are all highly active. The 2-pyridyl compound **16** (IC_50_=33 nm) doubles the potency when compared with the sulfide linker analogue **13**, whereas 4-pyridyl derivative **18** (IC_50_=15 nm) displays a fourfold increase in inhibitory activity and is one of the most potent compounds in this series. Even the 6-methyl-substituted compound **17** retains the activity with an IC_50_ value of 24 nm. The trend of a sulfoxide linker compound normally exhibiting better activity than the corresponding sulfide analogue was also observed in our previous studies,[41, 42] suggesting the possibility of the oxygen on the sulfur forming added interactions with the protein or altering the geometry of the molecule placing the adamantyl and/or the aromatic ring in a preferred position for binding in the active site. Surprisingly, a detrimental effect was found when the sulfoxide was replaced with a sulfone group. Compounds **19**–**21** lose about 6–15-fold activity compared with their sulfoxide linker counterparts, indicating that the extra oxygen is not favoured for inhibition with such a structural combination and geometry.

**Table 2:** Cellular 11β-HSD1 inhibition by compounds **13**–**21**

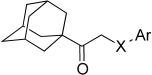			

Compd	X	Ar[a]	IC_50_[b] [nm]
**13**	S	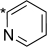	66
**14**	S	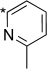	70 %[c]
**15**	S	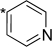	62
**16**	SO	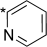	33
**17**	SO	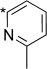	24
**18**	SO	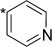	15
**19**	SO_2_	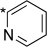	211
**20**	SO_2_	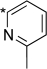	375
**21**	SO_2_	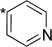	83

[a] * Indicates point of attachment.

[b] IC_50_ values are reported as the mean value of three measurements with variance less than 20 %.

[c] Percent inhibition measured at 1 μm and reported as the mean value of two measurements.

Alteration of the substituent on the 2-pyridyl ring was explored to investigate if a hydrogen-bonding group on the aromatic ring may potentially improve inhibition (Table 3). The substitution of a trifluoro group at the 5-position (**22**, IC_50_=3380 nm) results in a disastrous 51-fold reduction in activity when compared with the unsubstituted derivative **13**. The 5-carboxylic acid substitution performs better than that of the trifluoro group, but still gives a compound fivefold less active (**23**, IC_50_=345 nm) in comparison with **13**. Converting the acid to different carboxamide groups gives mixed results; while the CONH_2_ or CONMe_2_ substitution generates compounds (**24**, IC_50_=95 nm; **27**, IC_50_=80 nm) at nearly the same level as unsubstituted **13**, the CONHMe or CHNHEt substitution only gives compounds (**25**, **26**) of poor activity. The results suggest that the subtle change of a steric and/or electronic effect around the aromatic ring may have a large impact on inhibitory activity.

**Table 3:** Cellular 11β-HSD1 inhibition by compounds **22**–**27**

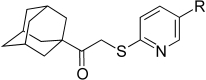		

Compd	R	IC_50_[a] [nm]
**22**	CF_3_	3380
**23**	COOH	345
**24**	CONH_2_	95
**25**	CONHMe	73 %[b]
**26**	CONHEt	7302
**27**	CONMe_2_	80

[a] IC_50_ values are reported as the mean value of three measurements with variance less than 20 %.

[b] Percent inhibition measured at 1 μm and reported as the mean value of two measurements.

The ethanone ether linker was further extended with an extra methylene unit to generate more flexible molecules (Table 4). The 2-pyridyl derivative **28** shows an impressive activity with an IC_50_ value of 48 nm. The 6-methyl substituent brings down the inhibition by twofold (**29**, IC_50_=99 nm), providing evidence that changing the substitution on the aromatic ring is not favoured. The 3-pyridyl derivative **31** (IC_50_=34 nm) displays the same level of activity as **28**, suggesting that the position of the nitrogen is not as restricted when combined with a more flexible linker. Attempts to achieve better activity with either the electron-donating methoxy group or the electron-withdrawing trifluoro group substitution were not successful, as compounds **30**, **32**–**34** all suffer some degree of potency loss when compared with nonsubstituted derivatives **28** or **31** (Table 4). Although compound **32** with a 4-methoxy group has an IC_50_ value of 100 nm, it is still threefold less active than **31**. To understand the importance of the ketone group, compounds **28** and **31** were transformed into their corresponding oxime analogues **35** and **36**, respectively; none of the oxime compound shows any significant inhibition at 1 μm. Reducing the ketone to an alcohol also results in a total loss of the activity (data not shown), suggesting that the carbonyl group is highly preferred in such a core structure.

**Table 4:** Cellular 11β-HSD1 inhibition by compounds **28**–**34**

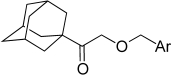		

Compd	Ar[a]	IC_50_[b] [nm]
**28**	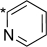	48
**29**	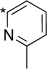	99
**30**	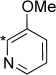	182
**31**	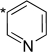	34
**32**	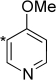	112
**33**	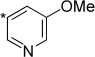	296
**34**	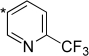	33 %[c]

[a] * Indicates point of attachment.

[b] IC_50_ values are reported as the mean value of three measurements with variance less than 20 %.

[c] Percent inhibition measured at 1 μm and reported as the mean value of two measurements.

Converting the oxygen linker of **28** or **31** to a sulfur linker leads to a compound twofold less active (**37**, IC_50_=86 nm; **38**, IC_50_=72 nm; Table 5). The 4-pyridyl compound **39** also exhibits similar potency with an IC_50_ value of 53 nm. However, it is interesting to note that sulfoxide linker analogues **40**–**42** regain activity by nearly twofold, following the same structure–activity relationship we observed previously. The 4-pyridyl derivative **42**, with an IC_50_ value of 26 nm, is the most potent compound in this series. It is also worth noting that that sulfone analogues **43**–**45** display high potency at nearly the same level as the sulfoxide compounds, especially 2-pyridyl and 4-pyridyl compounds (**43**, IC_50_=34 nm; **45**, IC_50_=34 nm); this is unlike the observation for pyridyl compounds with the short version of ethanone sulfone linker, which show reduced activity compared with the sulfoxide compounds (Table 2). It is assumed that even for compounds with a sulfone group linker, the molecular flexibility gained from the extended linker makes the adamantyl and/or aromatic moiety adapt to more preferred positions in the binding site.

**Table 5:** Cellular 11β-HSD1 inhibition by compounds **37**–**45**

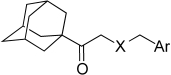			

Compd	X	Ar[a]	IC_50_[b] [nm]
**37**	S	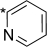	86
**38**	S	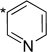	72
**39**	S	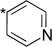	53
**40**	SO	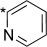	47
**41**	SO	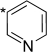	46
**42**	SO	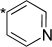	26
**43**	SO_2_	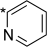	34
**44**	SO_2_	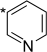	91
**45**	SO_2_	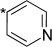	34

[a] * Indicates point of attachment.

[b] IC_50_ values are reported as the mean value of three measurements with variance less than 20 %.

The effect of a substituent on the pyridine motif was again investigated for the compounds with an extended linker (Table 6). The 5-methoxy-substituted compound **47** shows moderate activity with an IC_50_ value of 510 nm. With an electron-withdrawing group at the 6-position, both compounds **48** and **49** are poor inhibitors. The only compound with an IC_50_ value ∼100 nm is the sulfoxide linked 6-chloropyridyl derivative **50** (Table 6).

**Table 6:** Cellular 11β-HSD1 inhibition by compounds **47**–**53**

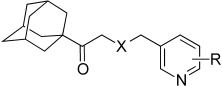			

Compd	X	R	IC_50_[a] [nm]
**47**	S	5-OMe	510
**48**	S	6-CF_3_	20 %[b]
**49**	S	6-Cl	31 %[b]
**50**	SO	6-Cl	117
**51**	SO_2_	5-OMe	67 %[b]
**52**	SO_2_	6-CF_3_	54 %[b]
**53**	SO_2_	6-Cl	903

[a] IC_50_ values are reported as the mean value of three measurements with variance less than 20 %.

[b] Percent inhibition measured at 1 μm and reported as the mean value of two measurements.

We also investigated the structure–activity relationship in the ethanone amide linker series (Table 7). The 2-pyridyl carboxamide **56** gives moderate potency with an IC_50_ value of 278 nm. Adding a 6-methyl group brings no change to the activity (**57**, IC_50_=294 nm); however, the 3-pyridyl derivative **58** (IC_50_=151 nm) is about twofold more active than **28**. Changing to a *para*-substituted pyridyl (**59**, IC_50_=239 nm) or inserting an extra methylene unit between the pyridyl ring and the amide group (**60**, IC_50_=193 nm; **61**, IC_50_=217 nm) gave compounds with only modest potency. Therefore, it is believed that the amide linker is not a suitable replacement for a sulfoxide or sulfone linker in this series.

**Table 7:** Cellular 11β-HSD1 inhibition by compounds **56**–**61**

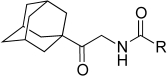		

Compd	Ar[a]	IC_50_[b] [nm]
**56**	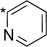	278
**57**	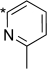	294
**58**	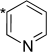	151
**59**	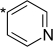	239
**60**	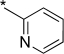	193
**61**	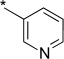	217

[a] * Indicates point of attachment.

[b] IC_50_ values are reported as the mean value of three measurements with variance less than 20 %.

To identify compounds with high 11β-HSD1 inhibitory activity against both human and mouse enzymes, we selected compounds with IC_50_ values below 50 nm on human 11β-HSD1 and examined their activity against the mouse enzyme. Compounds **16**, **28**, **31** and **43** all exhibit high potency against the mouse enzyme at the same level as that of the human enzyme with IC_50_ values below 50 nm in both cases, making them suitable candidates for further in vivo study in mouse models. On the other hand, compounds **12**, **40**–**42** and **45** all display significantly reduced activity against the mouse enzyme with IC_50_ values ranging from 150 to 250 nm. Although compounds **17** and **18** are three- to fourfold less active on the mouse enzyme, they still retain relatively high potency against both species (Table 8). The potent compounds selected for evaluation against mouse 11β-HSD1 (Table 8) were also tested for their inhibition against human 11β-HSD2 and 17β-HSD1 (data not shown). The results indicate that all these compounds are inactive at a concentration of 10 μm, and therefore they are regarded as highly selective 11β-HSD1 inhibitors.

**Table 8:** Comparison of cellular 11β-HSD1 inhibition of human and mouse enzymes[a]

Compd	IC_50_ (human) [nm]	IC_50_ (mouse) [nm]
**12**	27	160
**16**	33	43
**17**	24	68
**18**	15	61
**28**	48	44
**31**	34	28
**40**	47	248
**41**	46	148
**42**	26	191
**43**	34	35
**45**	34	150

[a] IC_50_ values are reported as the mean value of three measurements with variance less than 20 %.

As adamantyl ethanone compounds are lipophilic in nature, their solubility in the aqueous phase is rather limited, which could cause formulation issues in in vivo studies. The pyridine ring in the core structure of our potent compounds can be transformed into a salt form with improved solubility. Compound **31** was converted to its hydrochloride salt form (**62**), which demonstrates enhanced water solubility (>4 mg mL^−1^) in a Na_2_HPO_4_ buffer solution with or without 5 % *N*,*N*-dimethylacetamide (DMA) as a cosolvent. Compound **62** displays an IC_50_ value of 141 nm in the cellular assay against human 11β-HSD1. The difference in activity between the parent compound and its salt form may be due to the dissociation rate in the assay buffer.

### Permeability, metabolic stability and CYP450 inhibition studies

Compound **31** was evaluated for its membrane permeability using the Caco-2 cell model. The apparent permeability coefficient (*P*_app_) from apical to basolateral side was measured at a concentration of 20 μm; the result indicates that the compound is of high permeability (*P*_app_(A>B)=6.3×10 ^−6^), suggesting that **31** may have a good oral absorption property.

Metabolic stability is a critical factor in the selection of compounds for further preclinical studies. Potent inhibitors **16, 28, 31** and **42** were incubated with human liver microsomes to evaluate their stability under such conditions (Table 9). Two compounds (**16** and **42**) with a sulfoxide linker underwent rapid metabolism when incubated at 37 °C with human liver microsomes in the presence of the cofactor NADPH. With a half-life (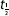) of only 20–30 min and a high intrinsic clearance rate, these compounds are not suitable for in vivo study. On the other hand, 2-pyridyl derivative **28** shows enhanced metabolic stability with a 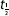 of 68 min and an intrinsic clearance of 8.9 μL min^−1^ mg^−1^. Furthermore, compound **31**, with the same ethanone ether linker and a 3-pyridyl ring, exhibits even greater metabolic stability, displaying a 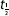 of more than 2 h and a clearance rate of 3.7 μL min^−1^ mg^−1^, indicating that metabolic stability may be achieved with this structural core. There were no metabolites identified for compounds **28** and **31** under such conditions.

**Table 9:** Metabolism studies in human liver microsomes[a]

Compd	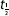 [min]	CL_int_ [μL min^−1^ mg^−1^]
**16**	20	31
**28**	68	8.9
**31**	128	3.7
**42**	34	19

[a] The parent compound was incubated at 37 °C with human liver microsomes in the presence of the cofactor NADPH for 40 min. Disappearance of the parent compound was monitored using HPLC. Half-life (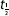) and intrinsic clearance (CL_int_) values were calculated accordingly.[44] Data are the mean value of two measurements.

Compounds **18**, **28, 31** and **43** were also examined for their inhibition of the key human cytochrome P450 enzymes: 1A2, 2C9, 2C19, 2D6, 3A4-BFC and 3A4-BQ (Table 10). These results may help identify potential problems with these compounds interfering with the metabolism of other drugs. All compounds tested display very weak activity against these cytochrome P450 enzymes, except compound **31** that shows a moderate inhibition of 3A4-BQ with an IC_50_ value of 1.6 μm, which is greater than 30-fold of its inhibitory activity against 11β-HSD1.

**Table 10:** Inhibition of human cytochrome P450 enzymes by selected compounds (**18**, **28**, **31**, **43**)

IC_50_ [μm][a]				
CYP450	**18**	**28**	**31**	**43**
1A2	>100	>100	23	36
2C9	>100	>100	>100	>100
2C19	>100	>100	46	24
2D6	>100	>100	ND	ND
3A4-BFC	12	20	68	>100
3A4-BQ	>100	78	1.6	24

[a] Values are reported as the mean of two measurements; ND: not determined.

### Docking studies

**Figure 2:**
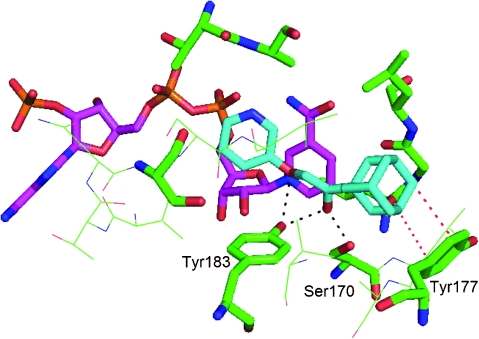
Best docking solution of compound **12** (in cyan) with the cofactor (in purple) and active residues (in green).

To investigate the potential binding mode of the inhibitor, compound **12** was docked into the X-ray crystal structure of human 11β-HSD1 (PDB: 2ILT[45]) using the GOLD docking program (v4.1). Ranked by the docking score, the top five predicted poses are almost identical. The pyridyl motif is placed close to the cofactor with the pyridyl nitrogen being 3.5 Å away from the NH_2_ group of the nicotinamide moiety. The carbonyl group possibly forms hydrogen-bond interactions with the hydroxy group of Ser 170 (2.3 Å) and/or Tyr 183 (2.6 Å). The ether oxygen could also form contacts with the protein through Tyr 183 (2.8 Å). The hydrophobic adamantyl group is predicted to stack with Tyr 177 at a distance of ∼3.5 Å (Figure 2).

**Figure 3:**
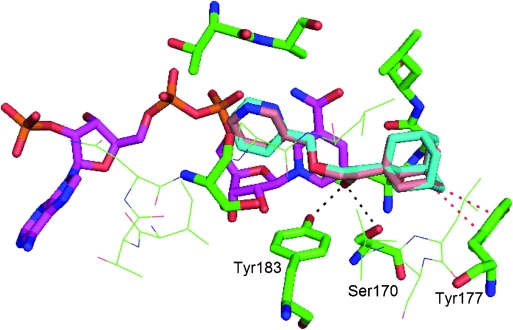
Best docking pose for compounds **28** (in cyan) and **31** (in buff) with the cofactor (in purple) and active residues (in green).

Docking studies with **28** and **31** reveal that the best poses of these two compounds overlap nicely with each other. The adamantyl moiety is predicted to form hydrophobic contacts with Tyr 177 in a distance of ∼3.5 Å in both cases. The carbonyl groups from **28** and **31** being 2.8 Å and 2.6 Å away from the hydroxy group of the catalytic residue Tyr 183, respectively, are able to act as hydrogen-bond acceptors. In addition, the hydroxy group of Ser 170 could form a hydrogen bond to the carbonyl group of **28** and **31** in a distance of 3.4 Å and 3.1 Å, respectively. The ether oxygen in the linker of **28** and **31** is placed 3.3 Å and 3.4 Å, respectively, away from the phenolic group on the catalytic residue Tyr 183, suggesting possible hydrogen-bond interactions between them (Figure 3).

**Figure 4:**
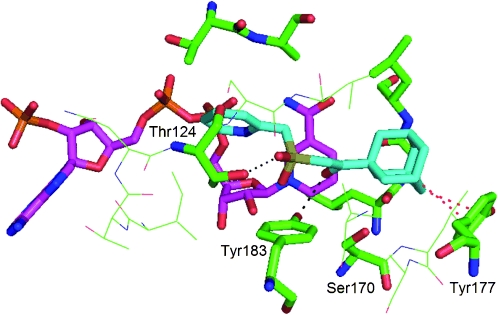
Best docking pose for compound **43** (in cyan) with the cofactor (in purple) and active residues (in green).

A docking study with compound **43** shows the highest ranking solution positioning the molecule in a pose similar to that of compounds **28** and **31.** The adamantyl motif is predicted to interact with the protein through hydrophobic interactions with Tyr 177 and Leu 217. The carbonyl group, being 2.7 Å away from Tyr 183, is most likely acting as a hydrogen-bond acceptor. One of the sulfone oxygen atoms is predicted to be close to the hydroxy group of residue Tyr 183 (2.7 Å) with which it may form a hydrogen bond. The other sulfone oxygen may be involved in a hydrogen bond to Thr 124 (3.1 Å) (Figure 4).

## Conclusions

In summary, adamantyl ethanone pyridyl derivatives have been identified as potent and selective inhibitors of human and mouse 11β-HSD1. Eleven compounds display high inhibitory activities against 11β-HSD1, with IC_50_ values below 50 nm when evaluated on a stably transfected HEK-293 cell line. They are also highly selective 11β-HSD1 inhibitors, with no activity against 11β-HSD2 and 17β-HSD1. Structure–activity relationship studies reveal that a pyridyl ring tethered to an adamantyl ethanone moiety through an oxygen, sulfoxide or sulfone linker represents a suitable pharmacophore for inhibitory activity. A substitution on the pyridyl ring normally results in a loss of activity, as most of the highly potent compounds in this series contain a unsubstituted pyridine ring. The inhibitors with a sulfoxide linker are consistently highly potent regardless of the linker length, suggesting important interactions of the S=O group in the enzyme pocket. Compounds **28** and **31**, with an ethanone ether linker, are not only potent 11β-HSD1 inhibitors of both the human and mouse enzyme, but are also relatively stable when incubated in human liver microsomes and show only very weak inhibition of key human CYP450 enzymes. Results from in vitro studies suggest that these compounds may be worthy of further preclinical investigation.

## Experimental Section

*Cellular 11β-HSD1 inhibition assay with scintillation proximity assay (SPA) protocol*:[46] Wild-type HEK293 cells lack endogenous 11β-HSD1 activity, and this cell line has been shown to be a suitable system for evaluating 11β-HSD1 activity after transfection with the plasmid for 11β-HSD1 or 11β-HSD2 expression.[47] The enzyme activity was determined by measuring the amount of tritiated product using a scintillation proximity assay (SPA). The high-throughput cell-based assays were conducted on the HEK293 cell line stably transfected with the *HSD11B1* gene using modified literature protocols. Cells were incubated in 96-well microplates in the presence of tritiated substrate, and the assay plates contained internal high and low controls to allow calculation of inhibition as a percentage. Each well of a 96-well culture plate was seeded with HEK293/HSD11B1 cells (2×10^4^ cells per well) in 100 μL medium. When the cell culture was 80 % confluent, the medium was removed from each well and replaced with 100 μL of fresh, serum-free, medium containing [^3^H]cortisone (10 μL of [^3^H]cortisone stock 51 ci mmol^−1^), and test compound in 1 % DMSO was added to each well. The final substrate concentration was ∼0.5 ci mmol^−1^. The control wells were also dispensed. The high control wells did not contain compound, while low controls did not contain cells. The plate was incubated at 37 °C for 2 h, after which, 50 μL of media was removed from each well and transferred to a microplate containing 100 μL of a preincubated mixture of anticortisol antibody and SPA bead. The mixture was incubated with gentle shaking until equilibrium was reached, before transferring to a scintillation counter to establish the enzyme activity in each sample.

**Docking study procedure**: Selected ligands were docked into the 11β-HSD1 protein X-ray crystal structure PBD: 2ILT[45] using the GOLD docking program (v4.1) with default settings in the presence of the cofactor. The binding site was defined as a sphere of 10 Å radius around the centroid of the ligand in the 2ILT structure. Each ligand was docked 25 times. The GOLDscore scoring function was used to rank the ligands in order of fitness.

### Chemistry

**General methods**: All chemicals were purchased from either Aldrich Chemical Co. (Gillingham, UK) or Alfa Aesar (Heysham, UK). All organic solvents of AR grade were supplied by Fisher Scientific (Loughborough, UK). Melting points were determined using a Stanford Research Systems Optimelt MPA100 and are uncorrected. Compounds in solid form were crystallised from CH_2_Cl_2_/EtOAc. Thin layer chromatography (TLC) was performed on precoated aluminium plates (Merck, silica gel 60 F_254_). Products were visualised by UV irradiation at 254 nm and by staining with 5 % *w*/*v* molybdophosphoric acid in EtOH, followed by heating. Flash column chromatography was performed on prepacked columns (RediSep RF) and gradient elution (solvents indicated in text) on the Combiflash RF system (Teledyne Isco). ^1^H NMR spectra were recorded on a Jeol Delta 270 mHz spectrometer. Chemical shifts (δ) are reported in parts per million (ppm) relative to tetramethylsilane (TMS) as an internal standard. LC/MS spectra were recorded on a Waters 2790 instrument using a Waters Symmetry C18 column (packing: 3.5 μm, 4.6 mm×75 mm) eluting with 10 % H_2_O/CH_3_OH (1 mL min^−1^), and detected with a ZQ MicroMass spectrometer and PDA detector using atmospheric pressure chemical ionisation (APCI) or electrospray ionisation (ESI). High-resolution mass spectra were recorded on a Bruker MicroTOF with ESI or at the EPSRC National Mass Spectrometry Service (Swansea, UK) with fast atom bombardment (FAB) using *m*-nitrobenzyl alcohol (NBA) as the matrix. HPLC was undertaken using a Waters 717 machine with Autosampler and PDA detector. The column used was a Waters Symmetry C18 (packing: 3.5 μm, 4.6 mm×150 mm) with an isocratic mobile phase consisting of H_2_O/CH_3_CN at a flow rate of 1.0 mL min^−1^.

**Method A: Synthesis of adamantyl ethanone pyridyl ether compounds 6**–**12**: A suspension of K_2_CO_3_ (2 equiv) in acetone (10 mL) was treated with the corresponding hydroxypyridine (1 equiv), followed by adamantan-1-yl bromomethyl ketone (1 equiv). The mixture was stirred at RT overnight. After quenching with water (20 mL), the mixture was extracted twice with EtOAc (30 mL). The organic phase was washed with brine, dried (MgSO_4_), filtered and concentrated in vacuo. The crude product was purified with flash chromatography (hexane/EtOAc or CH_2_Cl_2_/EtOAc; gradient elution).

**1-(Adamantan-1-yl)-2-[(6-methylpyridin-2-yl)oxy]ethan-1-one (6)**: A white solid (69 %); mp: 85–87 °C; ^1^H NMR (270 mHz, CDCl_3_): *δ*=1.68–1.81 (m, 6 H), 1.94 (br s, 6 H), 2.05 (br s, 3 H), 2.32 (s, 3 H), 5.10 (s, 2 H), 6.45 (dd app, *J=*7.2, 3.9 Hz, 2 H), 7.42 ppm (t, *J=*7.7 Hz, 1 H); LC/MS (APCI): *m*/*z*: 286 [*M*+H]^+^; HRMS (FAB): *m*/*z* [*M*+H]^+^ calcd for C_18_H_24_NO_2_: 286.1807, found: 286.1804; HPLC: *t*_R_=8.9 min (>99 %) in 10 % H_2_O/CH_3_CN.

**1-(Adamantan-1-yl)-2-[(6-chloropyridin-2-yl)oxy]ethan-1-one (7)**: A white solid (99 %); mp: 90–93 °C; ^1^H NMR (270 mHz, CDCl_3_): *δ*=1.65–1.82 (m, 6 H), 1.93 (d, *J=*2.5 Hz, 6 H), 2.06 (br s, 3 H), 5.10 (s, 2 H), 6.76 (d, *J=*8.3 Hz, 1 H), 6.85 (d, *J=*7.5 Hz, 1 H), 7.51 ppm (t, *J=*8.3 Hz, 1 H); LC/MS (APCI): *m*/*z*: 328 [*M*+Na]^+^; HRMS (FAB): *m*/*z* [*M*+H]^+^ calcd for C_17_H_21_ClNO_2_: 306.1261, found: 306.1261; HPLC: *t*_R_=4.0 min (>99 %) in 10 % H_2_O/CH_3_CN.

**1-(Adamantan-1-yl)-2-{[5-(trifluoromethyl)pyridin-2-yl]oxy}ethan-1-one (8)**: A white solid (90 %); mp: 102–104 °C; ^1^H NMR (270 mHz, CDCl_3_): *δ*=1.65–1.83 (m, 6 H), 1.93 (d, *J=*2.7 Hz, 6 H), 2.08 (br s, 3 H), 4.85 (s, 2 H), 6.58 (br d, *J=*10.4 Hz, 1 H), 7.40–7.48 ppm (m, 2 H); LC/MS (APCI): *m*/*z* 338 [*M*−H]^+^; HRMS (FAB): *m*/*z* [*M*+H]^+^ calcd for C_18_H_21_F_3_NO_2_ 340.1524, found: 340.1521; HPLC: *t*_R_=2.2 min (99 %) in 10 % H_2_O/CH_3_CN.

**1-(Adamantan-1-yl)-2-[(5-chloropyridin-3-yl)oxy]ethan-1-one (9)**: An off-white solid (76 %); mp: 127–128 °C; ^1^H NMR (270 mHz, CDCl_3_): *δ*=1.63–1.82 (m, 6 H), 1.88 (d, *J=*2.5 Hz, 6 H), 2.07 (br s, 3 H), 4.89 (s, 2 H), 7.06–7.12 (m, 1 H), 8.12 (d, *J=*2.2 Hz, 1 H), 8.17 ppm (d, *J=*1.4 Hz, 1 H); LC/MS (APCI): *m*/*z:* 328 [*M*+Na]^+^; HRMS (FAB): *m*/*z* [*M*+H]^+^ calcd for C_17_H_21_ClNO_2_: 306.1261, found: 306.1244; HPLC: *t*_R_=2.8 min (>99 %) in 10 % H_2_O/CH_3_CN.

**1-(Adamantan-1-yl)-2-[(2-chloropyridin-3-yl)oxy]ethan-1-one (10)**: An off-white solid (98 %); mp: 125–128 °C; ^1^H NMR (270 mHz, CDCl_3_): *δ*=1.63–1.82 (m, 6 H), 1.88 (d, *J=*2.8 Hz, 6 H), 2.05 (br s, 3 H), 4.94 (s, 2 H), 6.92 (dd, *J=*8.0, 1.4, Hz, 1 H), 7.10 (dd, *J=*8.0, 4.7 Hz, 1 H), 7.96 (dd, *J=*4.7, 1.4 Hz, 1 H); LC/MS (APCI): *m*/*z:* 328 [*M*+Na]^+^; HRMS (FAB): *m*/*z* [*M*+H]^+^ calcd for C_17_H_21_ClNO_2_: 306.1261, found: 306.1246; HPLC: *t*_R_=2.4 min (>99 %) in 10 % H_2_O/CH_3_CN.

**1-(Adamantan-1-yl)-2-[(6-methylpyridin-3-yl)oxy]ethan-1-one (11)**: An off-white solid (80 %); mp: 86–87 °C; ^1^H NMR (270 mHz, CDCl_3_): *δ*=1.67–1.82 (m, 6 H), 1.90 (br d, *J=*3.0 Hz, 6 H), 2.02–2.11 (br s, 3 H), 2.47 (s, 3 H), 4.87 (s, 2 H), 7.00–7.10 (m, 2 H), 8.12 ppm (dd, *J=*2.5, 1.2 Hz, 1 H); LC/MS (APCI): *m*/*z*: 286 [*M*+H]^+^; HRMS (FAB): *m*/*z* [*M*+H]^+^ calcd for C_18_H_24_NO_2_: 286.1807, found: 286.1800; HPLC: *t*_R_=6.2 min (97 %) in 10 % H_2_O/CH_3_CN.

**1-(Adamantan-1-yl)-2-(pyridin-3-yloxy)ethan-1-one (12)**: An off-white solid (78 %); mp: 73–75 °C; ^1^H NMR (270 mHz, CDCl_3_): *δ*=1.75–1.82 (m, 6 H), 1.92 (d, *J*=2.7 Hz, 6 H), 2.08 (br s, 3 H), 4.91 (s, 2 H), 7.10–7.21 (m, 2 H), 8.22 (dd, *J*=4.6, 1.3 Hz, 1 H), 8.26 ppm (d, *J*=2.7 Hz, 1 H); LC/MS (ESI): *m*/*z*: 270 [*M*−H]^+^; HRMS (ESI): *m*/*z* [*M*+H]^+^ calcd for C_17_H_22_NO_2_: 272.1651, found: 272.1672; HPLC: *t*_R_=2.4 min (97 %) in 10 % H_2_O/CH_3_CN.

**Method B: Synthesis of the adamantyl ethanone sulfanyl derivatives 13**–**15**: A solution of adamantan-1-yl bromomethyl ketone (1 equiv) in CH_3_CN (10 mL) was treated with the corresponding mercaptan (1.1 equiv), followed by Et_3_N (3 equiv). The mixture was stirred at RT overnight. 2-Chloro-tritylchloride resin (1.1 equiv, 1.6 mmol g^−1^) was added and the mixture was stirred for 2 h, filtered and concentrated in vacuo. Purification using flash chromatography (hexane/EtOAc or CH_2_Cl_2_/EtOAc; gradient elution) gave the target compound.

**1-(Adamantan-1-yl)-2-(pyridin-2-ylsulfanyl)ethan-1-one (13)**: A white solid (99 %); mp: 60–61 °C; ^1^H NMR (270 mHz, CDCl_3_): *δ*=1.68–1.79 (m, 6 H), 1.94 (d, *J*=2.7 Hz, 6 H), 2.07 (br s, 3 H), 4.23 (s, 2 H), 6.93 (ddd, *J*=7.4, 4.9, 1.0 Hz, 1 H), 7.21 (dt, *J*=8.2, 1.0 Hz, 1 H), 7.44 (ddd, *J*=9.2, 7.4, 2.0 Hz, 1 H), 8.32 (dq, *J*=4.9, 1.0 Hz, 1 H); LC/MS (ESI): *m*/*z*: 288 [*M*+H]^+^; HRMS (ESI): *m*/*z* [*M*+H]^+^ calcd for C_17_H_22_NOS: 288.1422, found: 288.1431; HPLC: *t*_R_=3.6 min (>99 %) in 10 % H_2_O/CH_3_CN.

**1-(Adamantan-1-yl)-2-[(6-methylpyridin-2-yl)sulfanyl]ethan-1-one (14)**: A white solid (89 %); mp: 116–117 °C; ^1^H NMR(270 mHz, CDCl_3_): *δ*=1.68–1.82 (m, 6 H), 1.95 (d, *J*=2.7 Hz, 6 H), 2.07 (br s, 3 H), 2.43 (s, 3 H), 4.22 (s, 2 H), 6.78 (d, *J*=7.9 Hz, 1 H), 7.03 (d, *J*=7.9 Hz, 1 H), 7.33 ppm (t, *J*=7.9 Hz, 1 H); LC/MS (ESI): *m*/*z*: 302 [*M*+H]^+^; HRMS (ESI): *m*/*z* [*M*+H]^+^ calcd for C_18_H_24_NOS: 302.1579, found: 302.1583; HPLC: *t*_R_=4.5 min (>99 %) in 10 % H_2_O/CH_3_CN.

**1-(Adamantan-1-yl)-2-(pyridin-4-ylsulfanyl)ethan-1-one (15)**: A white solid (86 %); mp: 114–115 °C; ^1^H NMR (270 mHz, CDCl_3_): *δ*=1.70–1.82 (m, 6 H), 1.90 (d, *J*=2.8 Hz, 6 H), 2.08 (br s, 3 H), 4.01 (s, 2 H), 7.08 (dd, *J*=4.7, 1.8 Hz, 2 H), 8.38 ppm (dd, *J*=4.7, 1.8 Hz, 2 H); LC/MS (ESI): *m*/*z*: 288 [*M*+H]^+^; HRMS (ESI): *m*/*z* [*M*+H]^+^ calcd for C_17_H_22_NOS: 288.1422, found: 288.1420; HPLC: *t*_R_=2.6 min (>99 %) in 10 % H_2_O/CH_3_CN.

**Method C: Synthesis of the adamantyl ethanone sulfoxide and sulfone derivatives 16**–**21**: A cold solution of the corresponding sulfanyl derivative (1 equiv) in CH_2_Cl_2_ (10 mL) was treated with *m*-CPBA (2.5 equiv) and stirred at −10 °C→0 °C for 1 h. The reaction was partitioned between CH_2_Cl_2_ and 5 % aq NaHCO_3_, then separated. The organic phase was washed with brine, dried (MgSO_4_), filtered and concentrated in vacuo. The sulfoxide and sulfone were separated using flash chromatography (EtOAc/CH_2_Cl_2_; gradient elution).

**1-(Adamantan-1-yl)-2-(pyridine-2-sulfinyl)ethan-1-one (16)**: A white solid (65 %); mp: 66–68 °C; ^1^H NMR (270 mHz, CDCl_3_): *δ*=1.58–1.76 (m, 6 H), 1.81 (d, *J*=2.7 Hz, 6 H), 2.04 (br s, 3 H), 4.05(d, *J*=15.6 Hz, 1 H), 4.32 (d, *J*=15.6 Hz, 1 H), 7.38 (m, 1 H), 7.90–8.20 (m, 2 H), 8.61 ppm (dq, *J*=5.1, 0.8 Hz, 1 H); LC/MS (ESI): *m*/*z*: 302 [*M*−H]^+^; HRMS (FAB): *m*/*z* [*M*+H]^+^ calcd for C_17_H_22_NO_2_S: 304.1371, found: 304.1366; HPLC: *t*_R_=2.2 min (>99 %) in 10 % H_2_O/CH_3_CN.

**1-(Adamantan-1-yl)-2-(pyridine-2-sulfonyl)ethan-1-one (19)**: A white solid (25 %); mp: 129–131 °C; ^1^H NMR (270 mHz, CDCl_3_): *δ*=1.54–1.76 (m, 12 H), 2.04 (br s, 3 H), 4.67 (s, 2 H), 7.53 (ddd, *J*=7.8, 4.9, 1.8 Hz, 1 H), 7.97 (td, *J*=7.7, 1.8 Hz, 1 H), 8.08 (dt, *J*=8.0, 1.0 Hz, 1 H), 8.70 ppm (dq, *J*=5.0, 0.8 Hz, 1 H); LC/MS (ESI): *m*/*z*: 318 [*M*−H]^+^; HRMS (ESI): *m*/*z* [*M*+H]^+^ calcd for C_17_H_22_NO_3_S: 320.1320, found: 320.1315; HPLC: *t*_R_=2.1 min (>99 %) in 10 % H_2_O/CH_3_CN.

**1-(Adamantan-1-yl)-2-(6-methylpyridine-2-sulfinyl)ethan-1-one (17)**: A white solid (69 %); mp: 75–76 °C; ^1^H NMR (270 mHz, CDCl_3_): *δ*=1.59–1.76 (m, 6 H), 1.81 (d, *J*=2.7 Hz, 6 H), 2.04 (br s, 3 H), 2.57 (s, 3 H), 4.03 (d, *J*=15.4 Hz, 1 H), 4.26 (d, *J*=15.4 Hz, 1 H), 7.21 (t, *J*=4.4 Hz, 1 H), 7.80 ppm (d, *J*=4.4 Hz, 2 H); LC/MS (ESI): *m*/*z*: 318 [*M*+H]^+^; HRMS (ESI): *m*/*z* [*M*+H]^+^ calcd for C_18_H_24_NO_2_S: 318.2518, found: 318.2515; HPLC: *t*_R_=2.5 min (>99 %) in 10 % H_2_O/CH_3_CN.

**1-(Adamantan-1-yl)-2-(6-methylpyridine-2-sulfonyl)ethan-1-one (20)**: A white solid (17 %); mp: 151–152 °C; ^1^H NMR (270 mHz, CDCl_3_): *δ*=1.56–1.72 (m, 6 H), 1.77 (d, *J*=2.7 Hz, 6 H), 2.05 (br s, 3 H), 2.60 (s, 3 H), 4.67 (s, 2 H), 7.35 (d, *J*=7.5 Hz, 1 H), 7.82 (t, *J*=7.4 Hz, 1 H), 7.89 ppm (d, *J*=7.6 Hz, 1 H); LC/MS (ESI): *m*/*z*: 334 [*M*+H]^+^; HRMS (ESI): *m*/*z* [*M*+H]^+^ calcd for C_18_H_24_NO_3_S: 334.1477, found: 334.1458; HPLC: *t*_R_=2.6 min (>99 %) in 10 % H_2_O/CH_3_CN.

**1-(Adamantan-1-yl)-2-(pyridine-4-sulfinyl)ethan-1-one (18)**: A white solid (77 %); mp: 136–138 °C; ^1^H NMR (270 mHz, CDCl_3_): *δ*=1.60–1.68 (m, 6 H), 1.74 (d, *J*=2.7 Hz, 6 H), 2.02 (br s, 3 H), 3.86 (d, *J*=15.7 Hz, 1 H), 4.21 (d, *J*=15.7 Hz, 1 H), 7.62 (dd, *J*=4.7, 1.3 Hz, 2 H), 8.78 ppm (dd, *J*=4.7, 1.4 Hz, 2 H); LC/MS (ESI): *m*/*z*: 304 [*M*+H]^+^; HRMS (ESI): *m*/*z* [*M*+H]^+^ calcd for C_17_H_22_NO_2_S: 304.1371, found: 304.1359; HPLC: *t*_R_=2.0 min (>99 %) in 10 % H_2_O/CH_3_CN.

**1-(Adamantan-1-yl)-2-(pyridine-4-sulfonyl)ethan-1-one (21)**: A white solid (16 %); mp: 143–144 °C; ^1^H NMR (270 mHz, CDCl_3_): *δ*=1.61–1.70 (m, 6 H), 1.74 (d, *J*=2.7 Hz, 6 H), 2.05 (br s, 3 H), 4.33 (s, 2 H), 7.79 (dd, *J*=4.7, 1.4 Hz, 2 H), 8.90 ppm (dd, *J*=4.7, 1.4 Hz, 2 H); LC/MS (ESI): *m*/*z:* 320 [*M*+H]^+^; HRMS (ESI): *m*/*z* [*M*+H]^+^ calcd for C_17_H_22_NO_3_S: 320.1320, found: 320.1304; HPLC: *t*_R_=2.1 min (>99 %) in 10 % H_2_O/CH_3_CN.

**Compounds 22–23 were synthesized using Method B**:

**1-(Adamantan-1-yl)-2-{[5-(trifluoromethyl)pyridin-2-yl]sulfanyl}ethan-1-one (22)**: A white solid (86 %); mp: 141–142 °C; ^1^H NMR (270 mHz, CDCl_3_): *δ*=1.69–1.80 (m, 6 H), 1.94 (d, *J*=2.7 Hz, 6 H), 2.08 (br s, 3 H), 4.26 (s, 2 H), 7.30 (t, *J*=8.6 Hz, 1 H), 7.64 (dd, *J*=8.5, 2.5 Hz, 1 H), 8.55 ppm (s, 1 H); LC/MS (ESI): *m*/*z*: 354 [*M*−H]^+^; HRMS (ESI): *m*/*z* [*M*+Na]^+^ calcd for C_18_H_20_F_3_NOSNa: 378.1115, found: 378.1090; HPLC: *t*_R_=6.6 min (>99 %) in 10 % H_2_O/CH_3_CN.

**6-{[2-(Adamantan-1-yl)-2-oxoethyl]sulfanyl}pyridine-3-carboxylic acid (23)**: A white solid (65 %); mp: 174–176 °C; ^1^H NMR (270 mHz, CDCl_3_): *δ*=1.65–1.80 (m, 6 H), 1.95 (d, *J*=2.7 Hz, 6 H), 2.00 (br s, 3 H), 4.28 (s, 2 H), 7.31 (dd, *J*=8.6, 0.7 Hz, 1 H), 8.06 (dd, *J*=8.6, 2.2 Hz, 1 H), 8.97 ppm (dd, *J*=2.2, 0.7 Hz, 1 H); LC/MS (ESI): *m*/*z*: 332 [*M*+H]^+^; HRMS (ESI): *m*/*z* [*M*+H]^+^ calcd for C_18_H_22_NO_3_S: 332.1320, found: 332.1302; HPLC: *t*_R_=1.6 min (97 %) in 10 % H_2_O/CH_3_CN.

**Method D: Synthesis of the carboxamide derivatives 24**–**27**: A solution of carboxylic acid **23** (1.0 equiv) in CH_2_Cl_2_ (5 mL) was treated with EDCI (1.2 equiv), HOBt (0.5 equiv), Et_3_N (1.2 equiv) and DMAP (catalytic amount) at RT. After stirring for 30 min, the amine (1.2 equiv) was added, and the reaction mixture was stirred overnight and then extracted twice with CH_2_Cl_2_. The organic phase was washed with 5 % aq NaHCO_3_ and brine, dried (MgSO_4_), filtered and concentrated in vacuo. The crude product was purified using flash chromatography with EtOAc/CH_2_Cl_2_ gradient elution.

**6-{[2-(Adamantan-1-yl)-2-oxoethyl]sulfanyl}pyridine-3-carboxamide (24)**: A white solid (55 %); mp: 172–174 °C; ^1^H NMR (270 mHz, CDCl_3_): *δ*=1.69–1.82 (m, 6 H), 1.95 (d, *J*=2.7 Hz, 6 H), 2.08 (br s, 3 H), 4.24 (s, 2 H), 5.98 (br s, 2 H), 7.26 (d, *J*=8.5 Hz, 1 H), 7.86 (dd, *J*=8.6, 2.5 Hz, 1 H), 8.72 ppm (d, *J*=2.2 Hz, 1 H); LC/MS (ESI): *m*/*z*: 331 [*M*+H]^+^; HRMS (ESI): *m*/*z* [*M*+H]^+^ calcd for C_18_H_23_N_2_O_2_S: 331.1480, found: 331.1464; HPLC: *t*_R_=2.2 min (99 %) in 10 % H_2_O/CH_3_CN.

**6-{[2-(Adamantan-1-yl)-2-oxoethyl]sulfanyl}-*N*-methylpyridine-3-carboxamide (25)**: A white solid (46 %); mp: 184–186 °C; ^1^H NMR (270 mHz, CDCl_3_): *δ*=1.63–1.80 (m, 6 H), 1.94 (d, *J*=2.7 Hz, 6 H), 2.07 (br s, 3 H), 2.97 (d, *J*=5.0 Hz, 3 H), 4.23 (s, 2 H), 6.22 (br s, 1 H), 7.22 (d, *J*=8.7 Hz, 1 H), 7.80 (dd, *J*=8.7, 1.6 Hz, 1 H), 8.66 ppm (d, *J*=1.6 Hz, 1 H); LC/MS (ESI): *m*/*z*: 345 [*M*+H]^+^; HRMS (ESI): *m*/*z* [*M*+H]^+^ calcd for C_19_H_25_N_2_O_2_S: 345.1637, found: 345.1623; HPLC: *t*_R_=2.4 min (99 %) in 10 % H_2_O/CH_3_CN.

**6-{[2-(Adamantan-1-yl)-2-oxoethyl]sulfanyl}-*N*-ethylpyridine-3-carboxamide (26)**: A white solid (81 %); mp: 136–137 °C; ^1^H NMR (270 mHz, CDCl_3_): *δ*=1.22 (t, *J*=7.2 Hz, 3 H), 1.68–1.79 (m, 6 H), 1.93 (d, *J*=2.7 Hz, 6 H), 2.07 (br s, 3 H), 3.46 (m, 2 H), 4.23 (s, 2 H), 6.15 (s, 1 H), 7.22 (dd, *J*=8.6, 1.0 Hz, 1 H), 7.80 (dd, *J*=8.2, 2.2 Hz, 1 H), 8.66 ppm (d, *J*=1.6 Hz, 1 H); LC/MS (ESI): *m*/*z*: 359 [*M*+H]^+^; HRMS (ESI): *m*/*z* [*M*+H]^+^ calcd for C_20_H_27_N_2_O_2_S: 359.1793, found: 359.1773; HPLC: *t*_R_=2.6 min (98 %) in 10 % H_2_O/CH_3_CN.

**6-{[2-(Adamantan-1-yl)-2-oxoethyl]sulfanyl}-*N,N*-dimethylpyridine-3-carboxamide (27)**: A white solid (70 %); mp: 63–65 °C; ^1^H NMR (270 mHz, CDCl_3_): *δ*=1.65–1.79 (m, 6 H), 1.94 (d, *J*=2.7 Hz, 6 H), 2.07 (br s, 3 H), 3.02 (s, 3 H), 3.07 (s, 3 H), 4.23 (s, 2 H), 7.24 (dd, *J*=8.3, 0.8 Hz, 1 H), 7.53 (dd, *J*=8.3, 2.2 Hz, 1 H), 8.41 ppm (dd, *J*=2.2, 0.8 Hz, 1 H); LC/MS (ESI): *m*/*z*: 359 [*M*+H]^+^; HRMS (ESI): *m*/*z* [*M*+H]^+^ calcd for C_20_H_27_N_2_O_2_S: 359.1793, found: 359.1778; HPLC: *t*_R_=2.6 min (99 %) in 10 % H_2_O/CH_3_CN.

**Method E: Synthesis of ethanone ether linker compounds 28**–**34**: A suspension of NaH (60 % in mineral oil, 1.2 equiv) in dry THF (5 mL) was treated with the pyridylmethanol (1.0 equiv) at 0 °C. After stirring for 30 min, 1-adamantyl bromomethyl ketone (1.1 equiv) was added in dry THF (5 mL). The reaction was stirred for 2 h at 0 °C then at RT overnight. After quenching with water, the mixture was extracted twice with Et_2_O, washed with water then brine, dried (MgSO_4_), filtered and concentrated in vacuo. The crude product was purified using flash chromatography (CH_2_Cl_2_/CH_3_OH; gradient elution).

**1-(Adamantan-1-yl)-2-(pyridin-2-ylmethoxy)ethan-1-one (28)**: A clear oil (64 %); ^1^H NMR (270 mHz, CDCl_3_): *δ*=1.60–1.80 (m, 6 H) 1.81 (d, *J=*2.7 Hz, 6 H), 2.01 (br s, 3 H), 4.43 (s, 2 H), 4.67 (s, 2 H), 7.13–7.21 (m, 1 H), 7.51 (d, *J=*7.6 Hz, 1 H), 7.69 (td, *J=*7.6, 1.7 Hz, 1 H), 8.53 ppm (dq, *J=*5.0, 0.8 Hz, 1 H); LC/MS (APCI): *m*/*z*: 286 [*M*+H]^+^; HRMS (FAB): *m*/*z* [*M*+H]^+^ calcd for C_18_H_24_NO_2_: 286.1807, found: 286.1796; HPLC: *t*_R_=5.6 min (>99 %) in 10 % H_2_O/CH_3_CN.

**1-(Adamantan-1-yl)-2-[(6-methylpyridin-2-yl)methoxy]ethan-1-one (29)**: An off-white semi-solid (43 %); ^1^H NMR (270 mHz, CDCl_3_): *δ*=1.60–1.80 (m, 6 H), 1.81 (d app, *J=*3.0 Hz, 6 H), 2.02 (br s, 3 H), 2.52 (s, 3 H), 4.42 (s, 2 H), 4.64 (s, 2 H), 7.42 (d app, *J=*7.4 Hz, 1 H), 7.31 (d app, *J=*7.7 Hz, 1 H), 7.58 ppm (t, *J=*7.7 Hz, 1 H); LC/MS (APCI): *m*/*z*: 300 [*M*+H]^+^; HRMS (FAB): *m*/*z* [*M*+H]^+^ calcd for C_19_H_26_NO_2_: 300.1964, found: 300.1950; HPLC: *t*_R_=5.9 min (>99 %) in 10 % H_2_O/CH_3_CN.

**1-(Adamantan-1-yl)-2-[(3-methoxypyridin-2-yl)methoxy]ethan-1-one (30)**: A yellow oil (66 %); ^1^H NMR (270 mHz, CDCl_3_): *δ*=1.55–1.78 (m, 6 H), 1.81 (d, *J=*2.7 Hz, 6 H), 2.01 (br s, 3 H), 3.82 (s, 3 H), 4.31 (s, 2 H), 4.55 (s, 2 H), 7.15 (dd, *J=*7.9, 2.0 Hz, 1 H), 7.27 (t, *J=*7.7 Hz, 1 H), 8.26 ppm (dd, *J=*7.9, 2.1 Hz, 1 H); LC/MS (APCI): *m*/*z*: 316 [*M*+H]^+^; HRMS (FAB): *m*/*z* [*M*+H]^+^ calcd for C_19_H_26_NO_3_: 316.1913, found: 316.1897; HPLC: *t*_R_=2.3 min (97 %) in 10 % H_2_O/CH_3_CN.

**1-(Adamantan-1-yl)-2-(pyridin-3-ylmethoxy)ethan-1-one (31)**: Off-white crystals (48 %); mp: 67–68 °C; ^1^H NMR (270 mHz, CDCl_3_): *δ*=1.58–1.78 (m, 6 H), 1.80 (d, *J=*3.0 Hz, 6 H), 2.01 (br s, 3 H), 4.34 (s, 2 H), 4.56 (s, 2 H), 7.27 (dd, *J=*7.9, 4.9 Hz, 1 H), 7.74 (td, *J=*7.9, 2.0 Hz, 1 H), 8.56 ppm (m, 2 H); LC/MS (APCI): *m*/*z*: 286 [*M*+H]^+^; HRMS (FAB): *m*/*z* [*M*+H]^+^ calcd for C_18_H_24_NO_2_: 286.1807, found: 286.1796; HPLC: *t*_R_=5.6 min (> 99 %) in 10 % H_2_O/CH_3_CN.

**1-(Adamantan-1-yl)-2-[(4-methoxypyridin-3-yl)methoxy]ethan-1-one (32)**: An off-white semi-solid (29 %); ^1^H NMR (270 mHz, CDCl_3_): *δ*=1.59–1.75 (m, 6 H), 1.77 (d, *J*=2.8 Hz, 6 H), 1.99 (br s, 3 H), 3.89 (s, 3 H), 4.26 (s, 2 H), 4.46 (s, 2 H), 6.71 (d, *J*=8.5 Hz, 1 H), 7.63 (dd, *J*=8.5, 2.5 Hz, 1 H), 8.06 ppm (d, *J*=2.5 Hz, 1 H); LC/MS (APCI): *m*/*z*: 316 [*M*+H]^+^; HRMS (FAB): *m*/*z* [*M*+H]^+^ calcd for C_19_H_26_NO_3_: 316.1913, found: 316.1923; HPLC: *t*_R_=2.9 min (97 %) in 10 % H_2_O/CH_3_CN.

**1-(Adamantan-1-yl)-2-[(5-methoxypyridin-3-yl)methoxy]ethan-1-one (33)**: A light yellow oil (43 %); ^1^H NMR (270 mHz, CDCl_3_): *δ*=1.81–1.56 (m, 6 H), 1.81 (d, *J=*2.7 Hz, 6 H), 2.02 (br s, 3 H), 3.85 (s, 3 H), 4.33 (s, 2 H), 4.56 (s, 2 H), 7.29 (s, 1 H), 8.14 (s, 1 H), 8.24 ppm (d, *J=*3.0 Hz, 1 H); LC/MS (APCI): *m*/*z*: 316 [*M*+H]^+^; HRMS (FAB): *m*/*z* [*M*+H]^+^ calcd for C_19_H_26_NO_3_: 316.1913, found: 316.1907; HPLC: *t*_R_=2.2 min (99 %) in 10 % H_2_O/CH_3_CN.

**1-(Adamantan-1-yl)-2-{[6-(trifluoromethyl)pyridin-3-yl]methoxy}ethan-1-one (34)**: A white solid (29 %); mp: 106–109 °C; ^1^H NMR (270 mHz, CDCl_3_): *δ*=1.58–1.80 (m, 6 H), 1.80 (d, *J=*2.7 Hz, 6 H), 2.01 (br s, 3 H), 4.38 (s, 2 H), 4.63 (s, 2 H), 7.65 (d, *J=*7.9 Hz, 1 H), 7.93 (dd, *J=*7.9, 1.4 Hz, 1 H), 8.65 ppm (br s, 1 H); LC/MS (APCI): *m*/*z*: 376.0 [*M*+Na]^+^; HRMS (FAB+): *m*/*z* [*M*+H]^+^ calcd for C_19_H_23_F_3_NO_2_: 354.1681, found: 354.1664; HPLC: *t*_R_=3.2 min (97 %) in 10 % H_2_O/CH_3_CN.

***N*****-[1-(Adamantan-1-yl)-2-(pyridin-2-ylmethoxy)ethylidene]hydroxylamine (35)**: A solution of **28** (108 mg, 0.38 mmol) in EtOH was treated with HONH_2_⋅HCl (53 mg, 0.76 mmol) followed by Et_3_N (0.106 mL, 0.76 mmol). The mixture was refluxed with 4 Å molecular sieves overnight, cooled to RT and partitioned between EtOAc and water. The organic phase was washed with brine, dried (MgSO_4_), filtered and concentrated in vacuo. Purification using flash chromatography (CH_2_Cl_2_/CH_3_OH; gradient elution) gave the product as a white solid (74 mg, 65 %); mp: 102–105 °C; ^1^H NMR (270 mHz, CDCl_3_): *δ*=1.60–1.78 (m, 6 H), 1.83 (d, *J=*2.5 Hz, 6 H), 2.00 (br s, 3 H), 4.37 (s, 2 H), 4.72 (s, 2 H), 7.16 (m, 1 H), 7.51 (d, *J=*7.9 Hz, 1 H), 7.68 (td, *J=*7.5, 1.1 Hz, 1 H), 8.53 (dd, *J=*4.9, 0.8 Hz, 1 H), 9.93 ppm (s, 1 H); LC/MS (APCI): *m*/*z*: 301 [*M*+H]^+^; HRMS (FAB): *m*/*z* [*M*+H]^+^ calcd for C_18_H_25_N_2_O_2_: 301.1916, found: 301.1929; HPLC: *t*_R_=2.1 min (98 %) in 10 % H_2_O/CH_3_CN.

***N*****-[1-(Adamantan-1-yl)-2-(pyridin-3-ylmethoxy)ethylidene]hydroxylamine (36)**: Prepared using the same method as for **35**. A white solid (85 mg, 81 %); mp: 115–117 °C; ^1^H NMR (270 mHz, CDCl_3_): *δ*=1.58–1.76 (6 H, m), 1.81 (6 H, d, *J=*2.5 Hz), 2.00 (3 H, br s), 4.28 (2 H, s), 4.57 (2 H, s), 7.22–7.32 (1 H, m), 7.71 (1 H, d, *J=*8.0 Hz), 8.52 (1 H, br d, *J=*3.8 Hz), 8.59 (1 H, s), 9.93 ppm (br s, 1 H, OH); LC/MS (APCI): *m*/*z*: 301 [*M*+H]^+^; HRMS (FAB): *m*/*z* [*M*+H]^+^ calcd for C_18_H_25_N_2_O_2_: 301.1916, found: 301.1926; HPLC: *t*_R_=2.5 min (97 %) in 10 % H_2_O/CH_3_CN.

**Method F: Synthesis of the adamantyl ethanone sulfanyl derivatives 37**–**39**: A solution of pyridinylmethyl carbamimidothioate dihydrochloride (480 mg, 2.0 mmol) in water (10 mL) was treated with NaOH (160 mg, 4.0 mmol). The mixture was stirred at 80 °C under N_2_ for 45 min, cooled to RT and added to a solution of **5** (514 mg, 2.0 mmol) in CH_3_CN/Et_3_N (3 mL/2 mL). The mixture was stirred at RT overnight, then partitioned between CH_2_Cl_2_ and water. The organic phase was washed brine, dried (MgSO_4_), filtered and concentrated. Purification using flash chromatography (EtOAc/hexane; gradient elution) gave the desired compound.

**1-(Adamantan-1-yl)-2-[(pyridin-2-ylmethyl)sulfanyl]ethan-1-one (37)**: An off-white solid (550 mg, 91 %); mp: 38–39 °C; ^1^H NMR (270 mHz, CDCl_3_): *δ*=1.61–1.75 (m, 6 H), 1.81 (d, *J*=2.7 Hz, 6 H), 2.02 (br s, 3 H), 3.38 (s, 2 H), 3.83 (s, 2 H), 7.15 (ddd, *J*=7.6, 4.8, 1.0 Hz, 1 H), 7.36 (dt, *J*=7.8, 1.0 Hz, 1 H), 7.63 (td, *J*=7.6, 1.7 Hz, 1 H), 8.54 ppm (dq, *J*=5.0, 1.0 Hz, 1 H); LC/MS (ESI): *m*/*z*: 302 [*M*+H]^+^; HRMS (ESI): *m*/*z* [*M*+H]^+^ calcd for C_18_H_24_NOS: 302.1579, found: 302.1585; HPLC: *t*_R_=2.8 min (99 %) in 10 % H_2_O/CH_3_CN.

**1-(Adamantan-1-yl)-2-[(pyridin-3-ylmethyl)sulfanyl]ethan-1-one (38)**: An off-white solid (320 mg, 53 %); mp: 45–47 °C; ^1^H NMR (270 mHz, CDCl_3_): *δ*=1.62–1.75 (m, 6 H), 1.80 (d, *J*=2.7 Hz, 6 H), 2.02 (br s, 3 H), 3.21 (s, 2 H), 3.71 (s, 2 H), 7.24 (dd, *J*=7.7, 4.7 Hz, 1 H), 7.69 (dt, *J*=7.7, 1.8 Hz, 1 H), 8.49 (dd, *J*=4.7, 1.7 Hz, 1 H), 8.53 ppm (d, *J*=2.2 Hz, 1 H); LC/MS (ESI): *m*/*z*: 302 [*M*+H]^+^; HRMS (ESI): *m*/*z* [*M*+H]^+^ calcd for C_18_H_24_NOS: 302.1579, found: 302.1583; HPLC: *t*_R_=2.7 min (99 %) in 10 % H_2_O/CH_3_CN

**1-(Adamantan-1-yl)-2-[(pyridin-4-ylmethyl)sulfanyl]ethan-1-one (39)**: A yellow oil (331 mg, 55 %); ^1^H NMR (270 mHz, CDCl_3_): *δ*=1.62–1.75 (m, 6 H), 1.78 (d, *J*=2.7 Hz, 6 H), 2.01 (br s, 3 H), 3.17 (s, 2 H), 3.66 (s, 2 H), 7.24 (d, *J*=5.1 Hz, 2 H), 8.53 ppm (d, *J*=5.1 Hz, 2 H); LC/MS (ESI): *m*/*z*: 302 [*M*+H]^+^; HRMS (ESI): *m*/*z* [*M*+H]^+^ calcd for C_18_H_24_NOS: 302.1579, found: 302.1577; HPLC: *t*_R_=3.1 min (97 %) in 10 % H_2_O/CH_3_CN.

**Compounds 40–45 were synthesized using Method C**:

**1-(Adamantan-1-yl)-2-[(pyridin-2-ylmethyl)sulfinyl]ethan-1-one (40)**: A white solid (52 %); mp: 87–88 °C; ^1^H NMR (270 mHz, CDCl_3_): *δ*=1.69–1.79 (m, 6 H), 1.80 (d, *J*=2.7 Hz, 6 H), 2.05 (br s, 3 H), 3.87 (d, *J*=15.9 Hz, 1 H), 4.02 (d, *J*=15.9 Hz, 1 H), 4.19 (d, *J*=12.9 Hz, 1 H), 4.36 (d, *J*=12.9 Hz, 1 H), 7.25 (ddd, *J*=7.6, 4.9, 0.9 Hz, 1 H), 7.35 (d, *J*=7.7 Hz, 1 H), 7.70 (td, *J*=7.6, 1.7 Hz, 1 H), 8.60 ppm (dq, *J*=5.0, 0.7 Hz, 1 H); LC/MS (ESI): *m*/*z*: 340 [*M*+Na]^+^; HRMS (FAB): *m*/*z* [*M*+H]^+^ calcd for C_18_H_24_NO_2_S: 318.1528, found: 318.1521; HPLC: *t*_R_=1.9 min (>99 %) in 10 % H_2_O/CH_3_CN.

**1-(Adamantan-1-yl)-2-[(pyridin-2-ylmethyl)sulfonyl]ethan-1-one (43)**: A white solid (22 %); mp: 116–117 °C; ^1^H NMR (270 mHz, CDCl_3_): *δ*=1.64–1.78 (m, 6 H), 1.83 (d, *J*=2.7 Hz, 6 H), 2.07 (br s, 3 H), 4.26 (s, 2 H), 4.70 (s, 2 H), 7.28 (ddd, *J*=7.7, 4.9, 1.2 Hz, 1 H), 7.42 (d, *J*=7.7 Hz, 1 H), 7.72 (td, *J*=7.6, 2.0 Hz, 1 H), 8.59 ppm (dq, *J*=4.9, 0.8 Hz, 1 H); LC/MS (ESI): *m*/*z*: 332 [*M*−H]^+^; HRMS (ESI): *m*/*z* [*M*+H]^+^ calcd for C_18_H_24_NO_3_S: 334.1477, found: 334.1470; HPLC: *t*_R_=2.1 min (>99 %) in 10 % H_2_O/CH_3_CN.

**1-(Adamantan-1-yl)-2-[(pyridin-2-ylmethyl)sulfinyl]ethan-1-one (41)**: A white solid (62 %); mp: 116–119 °C; ^1^H NMR (270 mHz, CDCl_3_): *δ*=1.62–1.81 (m, 12 H), 2.05 (br s, 3 H), 3.56 (d, *J*=16.0 Hz, 1 H), 3.93 (d, *J*=16.0 Hz, 1 H), 4.02 (d, *J*=14.0 Hz, 1 H), 4.25 (d, *J*=14.0 Hz, 1 H), 7.32 (dd, *J*=7.9, 4.9 Hz, 1 H), 7.69 (dt, *J*=7.9, 2.2 Hz, 1 H), 8.49 (d, *J*=1.9 Hz, 1 H), 8.61 ppm (dd, *J*=4.9, 1.7 Hz, 1 H); LC/MS (ESI): *m*/*z*: 316 [*M*−H]^+^; HRMS (ESI): *m*/*z* [*M*+H]^+^ calcd for C_18_H_24_NO_2_S: 318.1528, found: 318.1514; HPLC: *t*_R_=1.8 min (>99 %) in 10 % H_2_O/CH_3_CN.

**1-(Adamantan-1-yl)-2-[(pyridin-2-ylmethyl)sulfonyl]ethan-1-one (44)**: A white solid (21 %); mp: 150–151 °C; ^1^H NMR (270 mHz, CDCl_3_): *δ*=1.63–1.73 (m, 6 H), 1.79 (d, *J*=2.7 Hz, 6 H), 2.08 (br s, 3 H), 3.89 (s, 2 H), 4.54 (s, 2 H), 7.34 (dd, *J*=7.9, 5.0 Hz, 1 H), 7.85 (dt, *J*=7.9, 1.7 Hz, 1 H), 8.63 (dd, *J*=5.0, 1.7 Hz, 1 H), 8.68 ppm (d, *J*=2.0 Hz, 1 H); LC/MS (ESI): *m*/*z*: 334 [*M*+H]^+^; HRMS (ESI): *m*/*z* [*M*+H]^+^ calcd for C_18_H_24_NO_3_S: 334.1477, found: 334.1475; HPLC: *t*_R_=1.9 min (>99 %) in 10 % H_2_O/CH_3_CN.

**1-(Adamantan-1-yl)-2-[(pyridin-2-ylmethyl)sulfinyl]ethan-1-one (42)**: A white solid (49 %); mp: 127–129 °C; ^1^H NMR (270 mHz, CDCl_3_): *δ*=1.62–1.76 (m, 12 H), 2.05 (br s, 3 H), 3.57 (d, *J*=16 Hz, 1 H), 3.96 (d, *J*=16 Hz, 1 H), 4.00 (d, *J*=14 Hz, 1 H), 4.24 (d, *J*=13 Hz, 1 H), 7.24 (dd, *J*=4.5, 1.6 Hz, 2 H), 8.63 ppm (dd, *J*=4.4, 1.6 Hz, 2 H); LC/MS (ESI): *m*/*z*: 318 [*M*+H]^+^; HRMS (FAB): *m*/*z* [*M*+H]^+^ calcd for C_18_H_24_NO_2_S: 318.1528, found: 318.1510; HPLC: *t*_R_=1.9 min (>99 %) in 10 % H_2_O/CH_3_CN.

**1-(Adamantan-1-yl)-2-[(pyridin-2-ylmethyl)sulfonyl]ethan-1-one (45)**: A white solid (26 %); mp: 119–123 °C; ^1^H NMR (270 mHz, CDCl_3_): *δ*=1.62–1.78 (m, 12 H), 2.07 (br s, 3 H), 3.88 (s, 2 H), 4.51 (s, 2 H), 7.42 (dd, *J*=4.4, 1.3 Hz, 2 H), 8.64 ppm (dd, *J*=4.4, 1.3 Hz, 2 H); LC/MS (ESI): *m*/*z*: 334 [*M*+H]^+^; HRMS (ESI): *m*/*z* [*M*+H]^+^ calcd for C_18_H_24_NO_3_S: 334.1477, found: 334.1443; HPLC: *t*_R_=2.0 min (98 %) in 10 % H_2_O/CH_3_CN.

**2-(Acetylsulfanyl)-1-(adamantan-1-yl)ethan-1-one (46)**: A solution of **5** (514 mg, 2.0 mmol) in CH_3_CN (25 mL) was treated with potassium thioacetate (251 mg, 2.2 mmol) and stirred at RT overnight. The reaction was diluted with water and extracted with EtOAc (3×30 mL). The organic phase was washed with brine, dried (MgSO_4_), filtered and concentrated in vacuo to give the desired product as an off-white semi-solid (468 mg, 93 %); ^1^H NMR (270 mHz, CDCl_3_): *δ*=1.66–1.88 (m, 6 H), 1.90 (d, *J*=2.7 Hz, 6 H), 2.32 (br s, 3 H), 2.35 (s, 3 H), 3.92 ppm (s, 2 H); LC/MS (ESI): *m*/*z*: 253 [*M*+H]^+^; HRMS (ESI): *m*/*z* [*M*+H]^+^ calcd for C_14_H_21_O_2_S: 253.1262, found: 253.1271; HPLC: *t*_R_=2.5 min (95 %) in 10 % H_2_O/CH_3_CN.

**Method G: Synthesis of the adamantyl ethanone sulfanyl derivatives 47–49**: A solution of **46** (1.0 equiv) in acetone (5 mL) was treated with 1 n NaOH (1.0 equiv) and stirred at RT under N_2_ for 1 h. The reaction was then treated with a solution of corresponding chloromethylpyridine (1.0 equiv) in CH_3_CN/Et_3_N (4 mL/2 mL), and the mixture was stirred at RT for overnight. The reaction was partitioned between EtOAc and water, and the organic phase was washed brine, dried (MgSO_4_), filtered and concentrated in vacuo. Purification using flash chromatography (EtOAc/hexane; gradient elution) gave the desired compound.

**1-(Adamantan-1-yl)-2-{[(5-methoxypyridin-3-yl)methyl]sulfan-yl}ethan-1-one (47)**: A clear oil (53 %); ^1^H NMR (270 mHz, CDCl_3_): *δ*=1.60–1.79 (m, 6 H), 1.80 (d, *J=*2.5 Hz, 6 H), 2.03 (br s, 3 H), 3.23 (s, 2 H), 3.70 (s, 2 H), 3.85 (s, 3 H), 7.21 (br s, 1 H), 8.12 (s, 1 H), 8.19 ppm (br d, *J*=2.5 Hz, 1 H); LC/MS (ESI): *m*/*z*: 332 [*M*+H]^+^; HRMS (ESI): *m*/*z* [*M*+H]^+^ calcd for C_19_H_26_NO_2_S: 332.1684, found: 332.1677; HPLC: *t*_R_=2.6 min (97 %) in 10 % H_2_O/CH_3_CN.

**1-(Adamantan-1-yl)-2-({[6-(trifluoromethyl)pyridin-3-yl]methyl}sulfanyl)ethan-1-one (48)**: A white solid (27 %); mp: 80–82 °C; ^1^H NMR (270 mHz, CDCl_3_): *δ*=1.58–1.80 (m, 6 H), 1.80 (d, *J=*2.7 Hz, 6 H), 2.02 (br s, 3 H), 3.18 (s, 2 H), 3.76 (s, 2 H), 7.62 (d, *J=*8.0 Hz, 1 H), 7.88 (dd, *J=*1.4, 8.0 Hz, 1 H), 8.65 ppm (br s,1 H); LC/MS (ESI): *m*/*z*: 368 [*M*−H]^+^; HRMS (ESI): *m*/*z* [*M*+H]^+^ calcd for C_19_H_23_F_3_NOS: 370.1452, found: 370.1436; HPLC: *t*_R_=3.4 min (98 %) in 10 % H_2_O/CH_3_CN.

**1-(Adamantan-1-yl)-2-{[(6-chloropyridin-3-yl)methyl]sulfanyl}ethan-1-one (49)**: An off-white solid (33 %); mp: 88–90 C; ^1^H NMR (270 mHz, CDCl_3_): *δ*=1.63–1.81 (m, 6 H), 2.03 (d, *J*=2.7 Hz, 6 H), 2.04 (br s, 3 H), 3.19 (s, 2 H), 3.68 (s, 2 H), 7.26 (d, *J*=8.3 Hz, 1 H), 7.68 (dd, *J*=8.3, 2.5 Hz, 1 H), 8.31 ppm (d, *J*=2.5 Hz, 1 H); LC/MS (ESI): *m*/*z:* 334 [*M*−H]^+^; HRMS (ESI): *m*/*z* [*M*+H]^+^ calcd for C_18_H_23_ClNOS: 336.1189, found: 336.1171; HPLC: *t*_R_=4.1 min (97 %) in 10 % H_2_O/CH_3_CN.

**Compounds 50–53 were synthesized using Method C**:

**1-(Adamantan-1-yl)-2-{[(6-chloropyridin-3-yl)methane]sulfinyl}ethan-1-one (50)**: A white solid (48 %); mp: 153–154 °C; ^1^H NMR (270 mHz, CDCl_3_): *δ*=1.63–1.77 (m, 12 H), 2.07 (br s, 3 H), 3.55 (d, *J*=16.0 Hz, 1 H), 3.96 (d, *J*=16.0 Hz, 1 H), 4.00 (d, *J*=14.0 Hz, 1 H), 4.24 (d, *J*=14.0 Hz, 1 H), 7.37 (d, *J*=8.2 Hz, 1 H), 7.68 (dd, *J*=8.2, 2.5 Hz, 1 H), 8.27 ppm (d, *J*=2.4 Hz, 1 H); LC/MS (ESI): *m*/*z*: 350 [*M*−H]^+^; HRMS (ESI): *m*/*z* [*M*+Na]^+^ calcd for C_18_H_22_ClNO_2_SNa: 374.0957, found: 374.0946; HPLC: *t*_R_=1.5 min (>99 %) in 10 % H_2_O/CH_3_CN.

**1-(Adamantan-1-yl)-2-{[(6-chloropyridin-3-yl)methane]sulfonyl}ethan-1-one (53)**: A white solid (24 %); mp: 159–161 °C; ^1^H NMR (270 mHz, CDCl_3_): *δ*=1.62–1.78 (m, 6 H), 1.79 (d, *J*=2.7 Hz, 6 H), 2.08 (br s, 3 H), 3.90 (s, 2 H), 4.52 (s, 2 H), 7.38 (d, *J*=8.3 Hz, 1 H), 7.82 (dd, *J*=8.3, 2.5 Hz, 1 H), 8.47 ppm (d, *J*=2.5 Hz, 1 H); LC/MS (ESI): *m*/*z*: 366 [*M*−H]^+^; HRMS (ESI): *m*/*z* [*M*+Na]^+^ calcd for C_18_H_22_ClNO_3_SNa: 390.0907, found: 390.0886; HPLC: *t*_R_=1.0 min (97 %) in 10 % H_2_O/CH_3_CN.

**1-(Adamantan-1-yl)-2-{[(5-methoxypyridin-3-yl)methane]sulfonyl}ethan-1-one (51)**: A white solid (51 %); mp: 152–154 °C; ^1^H NMR (270 mHz, CDCl_3_): *δ*=1.60–1.78 (m, 6 H), 1.78 (d, *J=*2.8 Hz, 6 H), 2.07 (br s, 3 H), 3.59 (s, 2 H), 3.87 (s, 3 H), 3.92 (s, 2 H), 7.35 (s, 1 H), 8.25 (s, 1 H), 8.36 ppm (d. *J=*2.5 Hz, 1 H); LC/MS (APCI): *m*/*z*: 364 [*M*+H]^+^; HRMS (FAB): *m*/*z* [*M*+H]^+^ calcd for C_19_H_26_NO_4_S: 364.1583, found: 364.1571; HPLC: *t*_R_=2.0 min (97 %) in 10 % H_2_O/CH_3_CN.

**1-(Adamantan-1-yl)-2-({[6-(trifluoromethyl)pyridin-3-yl]methyl}sulfonyl)ethan-1-one (52)**: A white solid (28 %); mp: 87–89 °C; ^1^H NMR (270 mHz, CDCl_3_): *δ*=1.76–1.60 (m, 6 H), 1.79 (d, *J=*2.7 Hz, 6 H), 2.08 (br s, 3 H), 3.93 (s, 2 H), 4.07 (s, 2 H), 7.72 (d, *J=*8.2 Hz, 1 H), 8.04 (dd, *J=*7.7, 1.7 Hz, 1 H), 8.80 ppm (br s,1 H); LC/MS (ESI): *m*/*z*: 400 [*M*−H]^+^; HRMS (ESI): *m*/*z* [*M*+H]^+^ calcd for C_19_H_23_F_3_NO_3_S: 402.1351, found: 402.1365; HPLC: *t*_R_=2.1 min (97 %) in 10 % H_2_O/CH_3_CN.

**1-(Adamantan-1-yl)-2-azidoethan-1-one (54)**: A cold solution of **5** (2.57 g, 10 mmol) in acetone (20 mL) was treated with NaN_3_ (780 mg, 12 mmol), and the mixture was stirred at 0 °C for 1 h and then at RT overnight. The reaction was then partitioned between EtOAc and water, and the organic phase was washed with brine, dried (MgSO_4_), filtered and concentrated in vacuo to give an off-white semi-solid (2.0 g, 91 %); ^1^H NMR (270 mHz, CDCl_3_): *δ*=1.65–1.77 (m, 6 H), 1.81 (d, *J*=2.8 Hz, 6 H), 2.04 (br s, 3 H), 4.04 ppm (s, 2 H); LC/MS (ESI): *m*/*z*: 242 [*M*+Na]^+^; HRMS (ESI): *m*/*z* [*M*+Na]^+^ calcd for C_12_H_17_N_3_NaO: 242.1269, found: 242.1257; HPLC: *t*_R_=2.7 min (98 %) in 10 % H_2_O/CH_3_CN.

**1-(Adamantan-1-yl)-2-aminoethan-1-one hydrochloride (55)**: A solution of **54** (1.9 g, 8.66 mmol) in CH_3_OH (80 mL) was treated with HCl (36 %, 1.8 mL), and the mixture was hydrogenated over 5 % Pd/C (300 mg) at atmospheric pressure for 6 h. The reaction was filtered through Celite and concentrated in vacuo. The white crystals obtained were filtered, washed with Et_2_O, and dried in vacuo (1.9 g, 95 %); ^1^H NMR (270 mHz, [D_6_]DMSO): *δ*=1.65–1.76 (m, 6 H), 1.79 (d, *J*=2.8 Hz, 6 H), 2.00 (br s, 3 H), 4.03 (s, 2 H), 8.11 ppm (br s, 2 H); LC/MS (ESI): *m*/*z*: 194 [*M*+H]^+^ (free base).

**Method H: Synthesis of the amide linker derivatives 56**–**61**: A solution of the corresponding pyridyl carboxylic acid or pyridyl acetic acid (1.0 equiv) in CH_2_Cl_2_ (5 mL) was treated with EDCI (1.2 equiv), HOBt (0.5 equiv), Et_3_N (2.5 equiv) and DMAP (catalytic amount) at RT. The mixture was stirred at RT for 30 min, and then compound **55** (1.2 equiv) was added, and the reaction was stirred overnight at RT. The mixture was partitioned between water and CH_2_Cl_2_, and the organic phase was washed with brine, dried (MgSO_4_), filtered and concentrated in vacuo. The crude product was purified using flash chromatography with EtOAc/CH_2_Cl_2_ gradient elution.

***N*****-[2-(Adamantan-1-yl)-2-oxoethyl]pyridine-2-carboxamide (56)**: A white solid (81 %); mp: 135–136 °C; ^1^H NMR (270 mHz, CDCl_3_): *δ*=1.65–1.82 (m, 6 H), 1.89 (d, *J*=2.7 Hz, 6 H), 2.06 (br s, 3 H), 4.43 (d, *J*=4.7 Hz, 2 H), 7.41 (ddd, *J*=7.4, 4.1, 1.1 Hz, 1 H), 7.82 (td, *J*=7.4, 1.5 Hz, 1 H), 8.15 (d, *J*=8.0 Hz, 1 H), 8.58 (dt, *J*=4.6, 1.0 Hz, 1 H), 8.70 ppm (br s, 1 H); LC/MS (ESI): *m*/*z*: 299 [*M*+H]^+^; HRMS (ESI): *m*/*z* [*M*+H]^+^ calcd for C_18_H_23_N_2_O_2_: 299.1760, found: 299.1741; HPLC: *t*_R_=2.3 min (99 %) in 10 % H_2_O/CH_3_CN.

***N*****-[2-(Adamantan-1-yl)-2-oxoethyl]-6-methylpyridine-2-carboxamide (57)**: A white solid (76 %); mp: 110–111 °C; ^1^H NMR (270 mHz, CDCl_3_): *δ*=1.65–1.80 (m, 6 H), 1.90 (d, *J*=2.6 Hz, 6 H), 2.07 (br s, 3 H), 2.57 (s, 3 H), 4.43 (d, *J*=4.7 Hz, 2 H), 7.26 (d, *J*=7.8 Hz, 1 H), 7.70 (t, *J*=7.7 Hz, 1 H), 7.95 (d, *J*=7.7 Hz, 1 H), 8.74 ppm (br s, 1 H); LC/MS (ESI): *m*/*z*: 313 [*M*+H]^+^; HRMS (ESI): *m*/*z* [*M*+H]^+^ calcd for C_19_H_25_N_2_O_2_: 313.1916, found: 313.1898; HPLC: *t*_R_=2.9 min (98 %) in 10 % H_2_O/CH_3_CN.

***N*****-[2-(Adamantan-1-yl)-2-oxoethyl]pyridine-3-carboxamide (58)**: A white solid (87 %); mp: 114–115 °C; ^1^H NMR (270 mHz, CDCl_3_): *δ*=1.65–1.82 (m, 6 H), 1.88 (d, *J*=2.7 Hz, 6 H), 2.08 (br s, 3 H), 4.43 (d, *J*=4.6 Hz, 2 H), 7.06 (br s, 1 H), 7.38 (dd, *J*=8.0, 4.7 Hz, 1 H), 8.11 (dt, *J*=8.0, 2.2 Hz, 1 H), 8.73 (dd, *J*=5.0, 1.8 Hz, 1 H), 9.03 ppm (d, *J*=2.2 Hz, 1 H); LC/MS (ESI): *m*/*z*: 299 [*M*+H]^+^; HRMS (ESI): *m*/*z* [*M*+H]^+^ calcd for C_18_H_23_N_2_O_2_: 299.1760, found: 299.1769; HPLC: *t*_R_=1.8 min (99 %) in 10 % H_2_O/CH_3_CN.

***N*****-[2-(Adamantan-1-yl)-2-oxoethyl]pyridine-4-carboxamide (59)**: A white solid (59 %); mp: 147–149 °C; ^1^H NMR (270 mHz, CDCl_3_): *δ*=1.69–1.82 (m, 6 H), 1.87 (d, *J*=2.7 Hz, 6 H), 2.08 (br s, 3 H), 4.42 (d, *J*=4.1 Hz, 2 H), 7.12 (br s, 1 H), 7.63 (dd, *J*=4.4, 1.6 Hz, 2 H), 8.75 ppm (dd, *J*=4.4, 1.6 Hz, 2 H); LC/MS (ESI): *m*/*z*: 299 [*M*+H]^+^; HRMS (ESI): *m*/*z* [*M*+H]^+^ calcd for C_18_H_23_N_2_O_2_: 299.1760, found: 299.1742; HPLC: *t*_R_=1.9 min (99 %) in 10 % H_2_O/CH_3_CN.

***N*****-[2-(Adamantan-1-yl)-2-oxoethyl]-2-(pyridin-2-yl)acetamide (60)**: A white solid (57 %); mp: 137–139 °C; ^1^H NMR (270 mHz, CDCl_3_): *δ*=1.62–1.82 (m, 12 H), 2.02 (br s, 3 H), 3.76 (s, 2 H), 4.23 (d, *J*=4.1 Hz, 2 H), 7.16–7.28 (m, 2 H), 7.63 (td, *J*=7.7, 1.6 Hz, 1 H), 7.85 (br s, 1 H), 8.61 ppm (m, 1 H); LC/MS (ESI): *m*/*z*: 313 [*M*+H]^+^; HRMS (ESI): *m*/*z* [*M*+H]^+^ calcd for C_19_H_25_N_2_O_2_: 313.1916, found: 313.1906; HPLC: *t*_R_=2.0 min (98 %) in 10 % H_2_O/CH_3_CN.

***N*****-[2-(Adamantan-1-yl)-2-oxoethyl]-2-(pyridin-3-yl)acetamide (61)**: A white solid (53 %); mp: 115–117 °C; ^1^H NMR (270 mHz, CDCl_3_): *δ*=1.61–1.82 (m, 12 H), 2.03 (br s, 3 H), 3.76 (s, 2 H), 4.21 (d, *J*=4.1 Hz, 2 H), 6.33 (br s, 1 H), 7.25 (dd, *J*=7.9, 4.9 Hz, 1 H), 7.65 (dt, *J*=6.1, 1.7 Hz, 1 H), 8.51 ppm (m, 2 H); LC/MS (ESI): *m*/*z*: 313 [*M*+H]^+^; HRMS (ESI): *m*/*z* [*M*+H]^+^ calcd for C_19_H_25_N_2_O_2_: 313.1916, found: 313.1913; HPLC: *t*_R_=1.7 min (98 %) in 10 % H_2_O/CH_3_CN.

**1-(Adamantan-1-yl)-2-(pyridin-3-ylmethoxy)ethan-1-one hydrochloride (62)**: A solution of **31** (428 mg, 1.5 mmol) in CH_2_Cl_2_ (1 mL) was treated with HCl (3 m in CH_3_OH, 2 mL, 6.0 mmol). The white solid was collected and washed with Et_2_O, and dried in vacuo (482 mg, 100 %); mp: 160–163 °C; ^1^H NMR (270 mHz, [D_6_]DMSO): *δ*=1.57–1.82 (m, 12 H), 2.05 (br s, 3 H), 4.10 (s, 2 H), 4.33 (s, 2 H), 7.38 (br d, *J=*6.9 Hz, 1 H), 7.82 ( br d, *J=*7.2 Hz, 1 H), 8.78 ppm (m, 2 H); LC/MS (ESI): *m*/*z* 286 [*M*+H]^+^ (free base); HRMS (ESI): *m*/*z* [*M*+H]^+^ (free base) calcd for C_18_H_24_NO_2_: 286.1807, found: 286.1796; HPLC: *t*_R_=2.1 min (>99 %) in 10 % H_2_O/CH_3_CN.
